# Multi-Cellular Rosettes in the Mouse Visceral Endoderm Facilitate the Ordered Migration of Anterior Visceral Endoderm Cells

**DOI:** 10.1371/journal.pbio.1001256

**Published:** 2012-02-07

**Authors:** Georgios Trichas, Aaron M. Smith, Natalia White, Vivienne Wilkins, Tomoko Watanabe, Abigail Moore, Bradley Joyce, Jacintha Sugnaseelan, Tristan A. Rodriguez, David Kay, Ruth E. Baker, Philip K. Maini, Shankar Srinivas

**Affiliations:** 1 Department of Physiology Anatomy & Genetics, University of Oxford, Oxford, United Kingdom; 2 Centre for Mathematical Biology, Mathematical Institute, University of Oxford, Oxford, United Kingdom; 3 Imperial College London, London, United Kingdom; 4 Oxford University Computing Laboratory, Oxford, United Kingdom; 5 Oxford Centre for Integrative Systems Biology, Department of Biochemistry, Oxford, United Kingdom; University of Kansas Medical Center, United States of America

## Abstract

The visceral endoderm (VE) is a simple epithelium that forms the outer layer of the egg-cylinder stage mouse embryo. The anterior visceral endoderm (AVE), a specialised subset of VE cells, is responsible for specifying anterior pattern. AVE cells show a stereotypic migratory behaviour within the VE, which is responsible for correctly orientating the anterior-posterior axis. The epithelial integrity of the VE is maintained during the course of AVE migration, which takes place by intercalation of AVE and other VE cells. Though a continuous epithelial sheet, the VE is characterised by two regions of dramatically different behaviour, one showing robust cell movement and intercalation (in which the AVE migrates) and one that is static, with relatively little cell movement and mixing. Little is known about the cellular rearrangements that accommodate and influence the sustained directional movement of subsets of cells (such as the AVE) within epithelia like the VE. This study uses an interdisciplinary approach to further our understanding of cell movement in epithelia. Using both wild-type embryos as well as mutants in which AVE migration is abnormal or arrested, we show that AVE migration is specifically linked to changes in cell packing in the VE and an increase in multi-cellular rosette arrangements (five or more cells meeting at a point). To probe the role of rosettes during AVE migration, we develop a mathematical model of cell movement in the VE. To do this, we use a vertex-based model, implemented on an ellipsoidal surface to represent a realistic geometry for the mouse egg-cylinder. The potential for rosette formation is included, along with various junctional rearrangements. Simulations suggest that while rosettes are not essential for AVE migration, they are crucial for the orderliness of this migration observed in embryos. Our simulations are similar to results from transgenic embryos in which Planar Cell Polarity (PCP) signalling is disrupted. Such embryos have significantly reduced rosette numbers, altered epithelial packing, and show abnormalities in AVE migration. Our results show that the formation of multi-cellular rosettes in the mouse VE is dependent on normal PCP signalling. Taken together, our model and experimental observations suggest that rosettes in the VE epithelium do not form passively in response to AVE migration. Instead, they are a PCP-dependent arrangement of cells that acts to buffer the disequilibrium in cell packing generated in the VE by AVE migration, enabling AVE cells to migrate in an orderly manner.

## Abstract 2

Modeling and experimental results suggest a role for Planar Cell Polarity-dependent multi-cellular rosette structures in ensuring correct epithelial cell migration in the mouse visceral endoderm.

## Author Summary

The mouse visceral endoderm (VE) is a simple epithelium in the egg cylinder stage mouse embryo. Many functions associated with epithelia require them to undergo extensive remodelling through changes in the shape and relative positions of constituent cells, a process about which we understand relatively little. The anterior visceral endoderm (AVE) is a specialized group of cells in the simple epithelium of the VE, and their stereotypic migratory behaviour is essential for establishing the orientation of the anterior-posterior axis in the early mouse embryo. We show that AVE migration is linked to changes in cell packing in the VE and an increase in “rosettes,” which are striking collections of five or more cells meeting at a central point. To probe the role of rosettes during AVE migration, we have developed a mathematical model of cell movement in the VE. Simulations suggest that rosettes are not essential for AVE migration, but are crucial for the *orderliness* of this migration. We also explored the role of Planar Cell Polarity (PCP) signalling, which is known to coordinate cell polarization and rearrangement in many different tissues. We find that mutants in which PCP signalling is disrupted have fewer rosettes, altered epithelial packing, and abnormal AVE migration. We suggest that rosettes in the mouse VE are a PCP-dependent arrangement of cells that act to buffer the disturbances in cell packing generated by AVE migration, thereby enabling AVE cells to migrate in an orderly manner.

## Introduction

Epithelia have structural and functional roles throughout embryonic development and adult life. Their organised, cohesive nature makes them ideal for lining structures and acting as selective barriers. Epithelia show distinct apical-basolateral polarity, with the apical domain characterised by junctional complexes that form tight junctions serving as a barrier to the flow of substances between cells. In addition, adherens junctions extend in a continuous belt around cells and provide structural integrity to epithelia. Many functions associated with epithelia during development, growth, disease, and repair require them to be highly dynamic whilst at the same time maintaining robust structural integrity. Most morphogenetic processes during development therefore involve extensive remodelling of epithelial tissues: branching morphogenesis in the developing kidneys, lungs, and mammary glands; development of sensory organs and ganglia from epithelial placodes; and the formation of the neural tube, to give just a few examples (reviewed in [1]–[5]).

The mouse visceral endoderm (VE) is an example of a simple epithelium with a critical developmental role. It covers the epiblast and extraembryonic ectoderm (ExE) of the egg-cylinder stage mouse embryo. Though the foetus is derived predominantly from the epiblast, it is cells of the VE that are responsible for specifying anterior pattern in the epiblast. The anterior visceral endoderm (AVE), a specialised subset of cells in the VE, is responsible for the correct orientation of the anterior-posterior axis in the mouse embryo (reviewed in [6]–[9]). At around 5½ days *post coitum* (dpc), cells at the distal tip of the VE differentiate to form the distinct subpopulation of the AVE, characterised by the expression of genetic markers such as *Hex*, *Lefty1*, and *Cer-1* [10]–[12]. The AVE migrates proximally in a unidirectional manner and then comes to an abrupt stop at the junction between the epiblast and ExE [13]. From this position, the AVE induces anterior pattern in the underlying epiblast by restricting expression of posterior markers to the opposite side of the epiblast cup [6],[14]. In mutants such as *Nodal* ^Δ600/LacZ^ and *Cripto* ^−/−^, the AVE is correctly induced at the distal tip of the egg-cylinder but fails to migrate, leading consequently to posterior markers in the epiblast being incorrectly localised. Such embryos show severe gastrulation defects and fail to develop further [15],[16].

The driving force for AVE migration remains unclear. Dkk1, a secreted inhibitor of Wnt signalling, is expressed just ahead of migrating AVE cells and has been shown to act as a guidance cue for the AVE [17]. A relatively higher level of cell proliferation in the posterior VE has been suggested to provide the initial displacement of AVE cells towards the anterior and possibly drive their directional migration [12], however more recent results suggest that the proliferation rate in the posterior VE is not higher than that in other regions of the VE and therefore unlikely to be involved in the movement of AVE cells [18]. Time-lapse microscopy of embryos carrying a Hex-GFP transgene that marks AVE cells shows that they actively migrate over a period of 4–5 h and are extremely dynamic, showing robust protrusive activity in the direction of motion [13]. Once AVE cells reach the border of the ExE, they abruptly cease proximal movement and instead start moving laterally along the boundary, as if in response to a barrier to migration. During this lateral movement, AVE cells show fewer or no protrusions [13],[19].

Recent reports have shown that the VE retains epithelial integrity during AVE migration [19],[20]. The tight and adherens junction markers ZO-1 and E-cadherin are present continuously along all cell borders of the entire VE at all stages of migration. In addition, AVE cells must migrate within the plane of the epithelium, rather than on top of other VE cells, because the VE remains a simple epithelium only one cell layer in thickness [13]. It would therefore seem necessary for AVE cells to negotiate their way through the VE without breaking epithelial integrity. This has been verified by time-lapse studies that show that AVE cell migration involves cell intercalation [19],[20].

Our time-lapse studies of the non-AVE cells of the VE show that the cells just ahead of (more proximal to) the migrating AVE show neighbour exchange during AVE migration [20]. Moreover, like the AVE, these cells too are unable to move beyond the boundary with the ExE. VE cells overlying the ExE (ExE-VE) show dramatically different behaviour in comparison to VE cells overlying the epiblast (Epi-VE). While the Epi-VE shows robust cell movement and intercalation, the ExE-VE in contrast is relatively static and shows very little cell mixing [20]. The barrier to AVE migration therefore appears to be a region of VE (the ExE-VE) that is non-permissive of the cell rearrangements required for AVE migration.

These two regions of the VE also show differences in localisation of the molecular motors F-actin and Myosin IIA, and the Planar Cell Polarity (PCP) signalling molecules Dishevelled-2 (Dvl-2) and Vangl2 [20]. PCP signalling coordinates cell polarisation and rearrangement across fields of cells in many different contexts, such as the compound-eye and wings of Drosophila, and the mammalian neural tube (reviewed in [21]–[23]). Morphogenetic cell movements in an epithelial context have been extensively studied in the Drosophila wing-disc and germband. Convergent extension movements in the germ band are brought about by junctional remodelling that results in T1-neighbour exchanges [24] and the formation and resolution of multi-cellular rosettes (five or more cells meeting at a point, Box 1) [25]. Germband extension is also characterised by an increase in the anisotropy of cells, initially regularly packed cells becoming increasingly disordered in their packing and shape [26].

**Box 1. Operational Definitions**

- *Rosettes*: groups or five or more cells within a simple epithelium that share a common central vertex.
- *Epithelial disequilibrium*: an epithelial state characterized by anisotropy of cell shapes, irregular cell packing, and reduction in the proportion of hexagonal cells.
- *Disordered AVE migration*: migration that results in a pattern of Hex-GFP AVE cells at 5.5 dpc that deviates from the stereotypic arrangement of a contained, coherent, bilaterally symmetrical patch of cells that does not extend onto the extra-embryonic ectoderm. Such deviations include whorl-like arrangements, AVE cells extending onto the extra-embryonic ectoderm, and AVE cells scattered and having a dispersed appearance. These phenotypes are not mutually exclusive and can occur together in the same embryo.

Epithelial tissues, including the mouse visceral endoderm, resemble two-dimensional networks of polygons [27],[28]. Vertex models, in which each cell is represented by a polygon with a limited set of properties, are therefore often used to simulate the tissue-level effects of forces and important cellular processes, such as growth, proliferation, and junctional rearrangements. Rauzi et al., for example, used a vertex model to show that tissue elongation can be driven by an anisotropy of cortical tension in combination with simple junctional rearrangements [29]. Aegerter-Wilmsen et al. meanwhile found that they were able to reproduce polygonal distributions in the Drosophila imaginal wing disc by including mechanical feedback as a regulator for cellular growth [30]. Several other authors have used vertex models to gain key insights into other biological phenomena [31]–[34].

Using a combination of mathematical modelling and experimental observations, we probe how the broader cell intercalation movements observed in the Epi-VE might influence AVE migration. By examining embryos at various stages of AVE migration and mutant embryos in which migration fails to take place, we show that AVE migration is specifically linked with changes in cell packing in the VE and an increase in multi-cellular rosette arrangements. To explore the role of rosettes during AVE migration, we have developed a mathematical model that simulates cell movements in the VE. This model extends previous vertex models by implementing an ellipsoidal surface to represent a realistic geometry for the mouse embryo. We also include a new type of junctional rearrangement, by allowing close vertices to join together, thus mimicking rosette formation. Simulations in which rosettes are allowed to form closely mimic experimentally observed AVE migratory behaviour. However, simulations in which rosettes are not allowed to form show abnormally disordered AVE migration (Box 1), suggesting that, while rosettes may not be essential for AVE migration, they are essential for the orderliness to this migration observed in actual embryos. These simulations closely recapitulate results from mutant embryos in which PCP signalling is disrupted and which have significantly reduced rosette numbers. AVE cells are still able to migrate to the anterior in these mutants, but do so in an abnormally dispersed, disordered manner. Our model and experimental observations together lead us to suggest that in the mouse VE, multi-cellular rosettes do not drive cell migration but rather buffer the disruption in cell packing arising from AVE migration, thereby enabling the AVE to migrate in an orderly manner.

## Results

### Cellular Packing within the Visceral Endoderm Changes with AVE Migration

To characterise in greater detail the changes in cellular packing in the VE that accompany and possibly influence AVE migration, we visualised apical boundaries of VE cells by staining fixed embryos for the tight junction marker ZO-1. We captured 3-D confocal image volumes of entire embryos and then opacity-rendered the image stacks. This provided volume renderings of entire embryos, so that the shape of individual cells of the surface VE and the junctions formed between them could be examined in the context of the cylindrical embryo as a whole. These experiments were performed with *Hex-GFP* transgenic embryos [35], in which the AVE is marked by GFP fluorescence.

**Figure 1:**
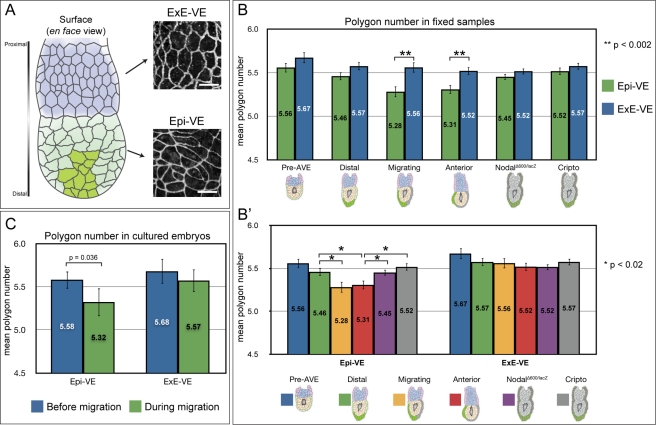
Cell packing within the visceral endoderm changes with AVE migration. (A) At left, diagram illustrating the mouse egg-cylinder, with the ExE-VE in blue (situated proximally) and Epi-VE in light green (situated distally). AVE cells are marked in dark green. At right, high magnification views of the VE of an egg-cylinder stage mouse embryo stained with ZO-1, showing differences in cell shape in the ExE-VE and Epi-VE. Scale bars = 25 µm. (B) The mean polygon number in the ExE-VE and Epi-VE at different wild-type stages (“pre-AVE”: before AVE induction, *n* = 337 Epi-VE and 231 ExE-VE cells from four embryos; “distal”: AVE at distal tip before migration, *n* = 497 Epi-VE and 396 ExE-VE cells from four embryos; “migrating”: AVE migrating, *n* = 300 Epi-VE and 236 ExE-VE cells from three embryos; and “anterior”: AVE finished proximal migration and moving laterally, *n* = 480 Epi-VE and 409 ExE-VE cells from three embryos) and in the AVE arrest mutants *Nodal^Δ600/lacZ^* (*n* = 724 Epi-VE and 565 ExE-VE cells from five embryos) and *Cripto* ^−/−^ (*n* = 605 Epi-VE and 598 ExE-VE cells from five embryos). At “migrating” and “anterior” stages, the mean polygon number in the Epi-VE is significantly lower than that in the ExE-VE. AVE arrest mutants isolated at a stage comparable to “anterior” embryos do not show this significant reduction in polygon number in the Epi-VE. (B′) The same polygon number data grouped according to the VE region. In the Epi-VE, mean polygon number in “migrating” and “anterior” embryos is significantly lower than that in “distal” embryos. The Epi-VE of AVE arrest mutants has a mean polygon number that is significantly different to that of stage matched “anterior” embryos but more similar to that of “distal” embryos. (C) Mean polygon numbers of Epi-VE (*n* = 31) and ExE-VE (*n* = 28) cells in five cultured embryos, measured at the start of and during AVE migration. The mean polygon number of the Epi-VE cells reduced significantly during migration, while that of the ExE-VE cells did not. *p* values shown on the graphs were determined using Student's *t* test.

In embryos in which the AVE had not yet commenced migration, cells were mostly regular in outline throughout the VE. In contrast, in embryos in which the AVE had migrated anteriorly, Epi-VE cells showed a great variety of shapes and irregular packing, though ExE-VE cells remained relatively regular in shape and packing (Figure 1A). This suggested the observed irregularities in cell shape might be related to the cell rearrangements in the Epi-VE that accompany AVE migration.

To quantify the differences in cell shape in the Epi-VE and ExE-VE at different stages of AVE migration, we counted the neighbours for each of the cells of the VE as a measure of the number of sides or *polygon number* of the cell [30]. Using the opacity renderings of fixed embryos, we manually identified each VE cell, noted whether it was located in the Epi-VE or ExE-VE and the number of cells that shared an edge with it. A hexagonal arrangement of cells (mean polygon number close to six) is considered to be the preferred or equilibrium packing of cells in an epithelium, and deviations from this are indicative of increased disequilibrium (Box 1) [26],[28].

We grouped embryos into four different stages of AVE migration using Hex-GFP fluorescence to determine whether the AVE had been induced and to what degree it had migrated. “Pre-AVE” embryos were those in which the AVE had not yet been induced. “Distal” embryos had the AVE induced at the distal tip, but it had not yet started migrating. In “migrating” embryos, the AVE was in the process of migrating, and in “anterior” embryos the AVE had reached the boundary of the ExE-VE (the proximal limit to migration) and was starting to spread laterally.

We compared polygon numbers in the ExE-VE and Epi-VE within each stage and found that in “pre-AVE” and “distal” embryos, the difference between mean polygon numbers in these two regions was not significant (*p*>0.07, Student's *t* test). However, in “migrating” and “anterior” embryos, the mean polygon number in the Epi-VE was significantly lower than that in the respective ExE-VE (*p*<0.002, Student's *t* test) (Figure 1B).

We compared polygon numbers across the different stages, and found no significant difference in the mean in the ExE-VE of the four stages (*p* = 0.25, ANOVA). However, there was a significant difference in the mean polygon numbers among the Epi-VE of the four stages (*p* = 0.0007, ANOVA). In pair-wise comparisons, the mean polygon number of the Epi-VE of “migrating” and “anterior” embryos were both significantly lower than that of the Epi-VE of “distal” embryos (*p* = 0.02, Student's *t* test) (Figure 1B′).

We next determined the frequency of the different polygon numbers in the Epi-VE and ExE-VE at the four stages in development. As with the mean polygon number, in “pre-AVE” and “distal” embryos, the distribution of polygon numbers in the Epi-VE was not significantly different from that in the respective ExE-VE (*p*>0.4, Kolmogorov-Smirnov test) (Figure S1A and B). By contrast, in “migrating” and “anterior” embryos, the distribution of polygon numbers in the Epi-VE was significantly different to that in the respective ExE-VE (*p*<0.02, Kolmogorov-Smirnov test), with a relatively higher proportion of four-sided cells (Figure S1C and D).

We compared polygon number frequencies in the Epi-VE across the four stages and found that it was not significantly different between “pre-AVE” and “distal” embryos (*p* = 0.5, Kolmogorov-Smirnov test) (Figure S2A). However, as with the mean polygon number, the frequencies of polygon numbers in the Epi-VE of both “migrating” and “anterior” embryos was significantly different from that in the Epi-VE of “distal” embryos (*p*<0.05, Kolmogorov-Smirnov test) (Figure S2B, C, and H). The Epi-VE of “migrating” and “anterior” embryos showed an increase in the proportion of four-sided cells at the expense of five- and six-sided cells as compared to “distal” embryos (Figure S2H), which would explain the significant reduction in mean polygon number in the Epi-VE of these stages.

The change we see in cell packing in the VE is localised to the region to which AVE migration is restricted (the Epi-VE) and to the stages during which AVE cells migrate (“migrating” and “anterior”). To verify if the change in packing of Epi-VE cells is linked specifically to AVE migration (as opposed, for instance, to the developmental stage of embryos), we examined *Nodal^Δ600/lacZ^* and *Cripto* ^−/−^ embryos, two mutants in which the AVE is correctly specified but fails to migrate. Embryos comparable to “anterior” stage wild-type embryos in size (*p* = 0.41, ANOVA) and shape (Figure S3) were dissected at 5.75 dpc and their polygon numbers determined.

We found that VE cell packing in both *Nodal^Δ600/lacZ^* and *Cripto* ^−/−^ embryos was more similar to that in “distal” embryos than to that in “anterior” embryos. In contrast to “anterior” embryos (but similar to “distal” embryos), neither *Nodal^Δ600/lacZ^* nor *Cripto* ^−/−^ embryos showed a significant difference in mean polygon number between their Epi-VE and respective ExE-VE (*p*>0.18, Student's *t* test) (Figure 1B). The frequencies of polygon numbers in the two regions were also similar (*p*>0.21, Kolmogorov-Smirnov test) (Figure S1E and F). Furthermore, when compared to the Epi-VE of “distal” embryos the Epi-VE of *Nodal^Δ600/lacZ^* and *Cripto* ^−/−^ embryos did not show a significant difference in mean polygon number (*p*>0.34, Student's *t* test) (Figure 1B′) or frequencies of polygon numbers (*p*>0.38, Kolmogorov-Smirnov test) (Figure S2D, E, and H). When compared to the Epi-VE of “anterior” embryos, the Epi-VE of stage-matched *Nodal^Δ600/lacZ^* and *Cripto* ^−/−^ embryos did show a significant difference in mean polygon number (*p*<0.02, Student's *t* test) (Figure 1B′) and frequencies of polygon numbers (*p*<0.03, Kolmogorov-Smirnov test) (Figure S2F, G, and H). Both mutants had a lower proportion of four-sided cells and higher proportion of six-sided cells as compared to “migrating” and “anterior” embryos (Figure S2H). These data all point to a specific link between AVE migration and changes in cell packing in the Epi-VE.

To confirm this is indeed the case, we determined the polygon number of VE cells in living embryos undergoing AVE migration. We visualised cell outlines in the VE of cultured embryos by differential interference contrast (DIC) time-lapse microscopy. Embryos were transgenic for Hex-GFP, enabling us to monitor AVE migration. We captured images from five focal planes at each time-point so cell outlines could be visualised unambiguously. We imaged embryos every 15 minutes to achieve sufficient time-resolution to follow individual cells from one time-point to the next. Due to the strong curvature of the surface of the embryo, only a relatively small portion of the surface VE could be viewed in focus. We analysed five embryos, in which we tracked a total of 31 Epi-VE and 28 ExE-VE cells during AVE migration, over an average period of 4 hours. We then compared the mean polygon number of these cells at the start of the experiment (when the AVE was at the distal tip of the embryo) with the mean polygon number of the *same* cells at the end of the experiment (when the AVE was in the process of migrating). The mean polygon number of the tracked Epi-VE cells was significantly lower during AVE migration compared to before the AVE had started migrating (*p* = 0.036, Student's *t* test on paired samples) (Figure 1C). The change in the mean polygon number of the “control” ExE-VE cells tracked during this same period was not significant (*p* = 0.238, Student's *t* test on paired samples). These results, together with the results from fixed wild-type and mutant embryos, strongly suggest migration of AVE cells is specifically accompanied by a reduction in mean polygon number in the Epi-VE and a shift away from the equilibrium cell packing arrangement.

### Multi-Cellular Rosettes in the VE Increase in Number During AVE Migration

**Figure 2:**
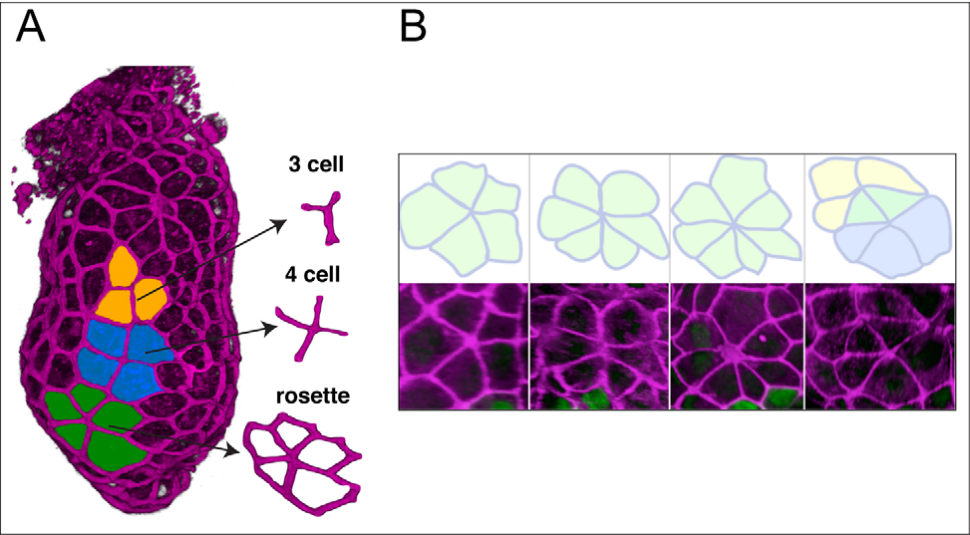
The VE contains multi-cellular rosettes. (A) A ZO-1 stained embryo in which cells are coloured in to illustrate the presence of junctions where three, four, or five cells meet at a point. (B) Rosettes are formed by five or more cells meeting at a point. A variety of rosettes are shown, including two that share some cells (last panel).

Renderings of the VE surface revealed a variety of junctions between cells. In addition to junctions where three cells meet at a point (typical of idealised hexagonal arrays of cells), we also frequently observed four-cell junctions and five or more VE cells meeting at a central point to form rosette arrangements (Figure 2A).

Rosettes typically comprised between five and seven cells, occasionally with one or two cells contributing to two distinct rosettes (Figure 2B). The majority of cells involved in rosettes were non-*Hex*-*GFP* expressing, though 8% of rosettes also included *Hex-GFP* cells (*n* = 51 rosettes). Examination of confocal sections and segmentation of rosettes to separately render individual cells in the context of the surrounding VE confirmed that rosettes are comprised of a single layer of cells, with all cells of the rosettes in contact with the epiblast (Movie S1).

Multi-cellular rosettes are characteristic intermediaries of long-range coordinated cell rearrangements during germband extension in *Drosophila* [25]. Together with the fundamental mechanism of T1 neighbour exchange [24], they are understood to drive convergent extension movements in the germband. To determine what role rosettes might play in the context of the mouse VE where no such convergent extension movements have been reported, we quantified rosette numbers in fixed embryos. As with our analysis of polygon numbers, we categorised embryos into four groups: “pre-AVE,” in which the AVE had not yet been induced; “distal,” in which the VE was at the distal tip, prior to migration; “migrating,” where the AVE was in the process of migration; and “anterior,” in which the AVE had reached the endpoint to proximal migration and had started moving laterally. We manually scored multi-cellular rosettes in opacity renderings of ZO-1 stained embryos for each category. To correct for any differences in the number of cells in the VE present and able to contribute to rosette formation, we divided the number of rosettes by the total number of VE cells for that embryo. We refer to this value as the rosette “density.” The average rosette density was then calculated for each group.

**Figure 3:**
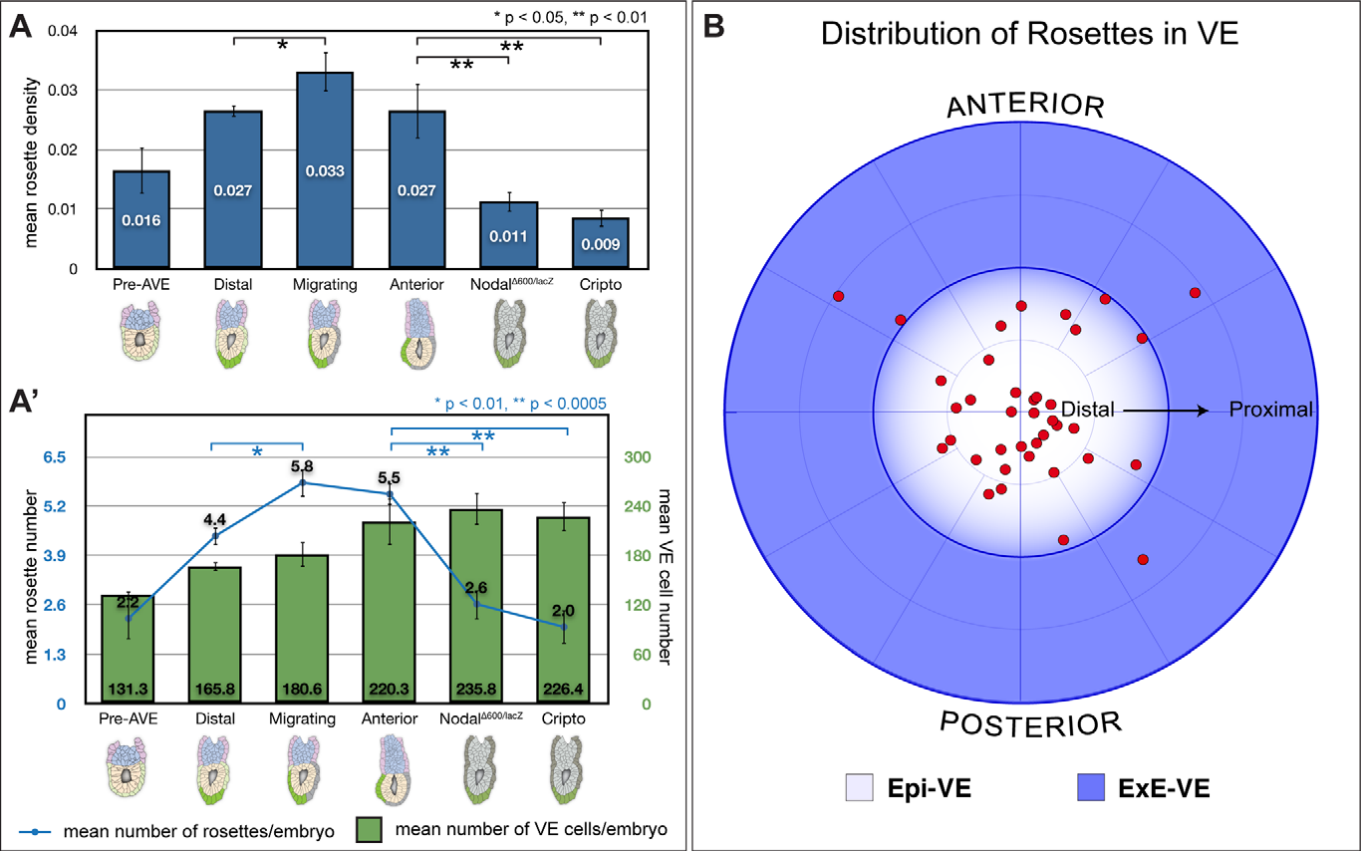
Quantitative characterisation of rosettes. (A) Rosette density (number of rosettes divided by total VE cell number) at different wild-type stages (“pre-AVE”: before AVE induction, *n* = 9; “distal”: AVE at distal tip before migration, *n* = 5; “migrating”: AVE migrating, *n* = 5; and “anterior”: AVE finished proximal migration and moving laterally, *n* = 4) and in the AVE arrest mutants *Nodal^Δ600/lacZ^* (*n* = 5) and *Cripto* ^−/−^ (*n* = 9). There is a significant increase in rosettes' density in “migrating” embryos as compared to “distal” embryos. The AVE arrest mutants *Nodal^Δ600/lacZ^* and *Cripto* ^−/−^ show significantly reduced rosette density compared to “migrating” and “anterior” embryos, suggestive of a direct link between rosettes and AVE migration. (A′) The same data as in (A), but depicted as mean number of rosettes per embryo (blue line), and mean number of VE cells per embryo (green bars) at the various stages. “Migrating” embryos have a comparable number of VE cells to “distal” embryos, but have significantly more rosettes, leading to an increase in rosette density. AVE arrest mutants have similar average VE cell numbers to stage matched “anterior” embryos, but show significantly fewer rosettes, leading to the reduced rosette density. (B) Polar plot showing distribution of rosettes in the VE of embryos. Migrating AVE cells were used to determine the anterior of embryos. Rosettes are localised predominantly to the Epi-VE. Within the Epi-VE, rosettes appear to be uniformly distributed with respect to the anterior-posterior axis (*n* = 39 rosettes from 7 embryos). *p* values shown on the graphs were determined using Student's *t* test.

Average rosette density was significantly different across the four groups (*p* = 0.025, ANOVA). We found a progressive increase in rosette density from “pre-AVE” to “distal” to “migrating” stages (Figure 3A). “Migrating” embryos had a significantly higher rosette density than “distal” and “pre-AVE” embryos (*p*<0.05, Student's *t* test). Rosettes' density decreased slightly from “migrating” to “anterior” stages, but not in a significant manner (*p* = 0.14, Student's *t* test).

The significant increase in rosette density during AVE migration suggests that rosette formation might be linked specifically to AVE migration. To confirm that this is indeed the case, we assessed rosette numbers in a double blind manner in *Nodal^Δ600/lacZ^* and *Cripto* ^−/−^ embryos, two mutants in which the AVE is correctly specified but fails to migrate [15],[16]. Embryos were dissected at 5.75 dpc, comparable to “anterior” stage wild-type embryos in size (Figure 3A′, *p* = 0.90, ANOVA) and shape (Figure S3) and rosette numbers determined. Mutants of both lines showed a significant reduction in rosette density when compared to both “anterior” and “migrating” stage embryos (Figure 3A) (*p*<0.01, Student's *t* test). Both mutants had a significant reduction in the average number of rosettes (Figure 3A′), leading to the observed reduction in rosette density.

To determine if rosettes are restricted to any one region of the VE, we plotted their distribution with respect to the future anterior and the boundary between the epiblast and ExE. Rosettes showed a strong bias in distribution with respect to the boundary between the epiblast and ExE, being located almost exclusively in the Epi-VE, the region to which AVE cell migration is restricted. Within the Epi-VE, they did not show any bias in distribution with respect to the presumptive anterior (Figure 3B).

### Rosettes Form by Cell Intercalation in the Epi-VE

**Figure 4:**
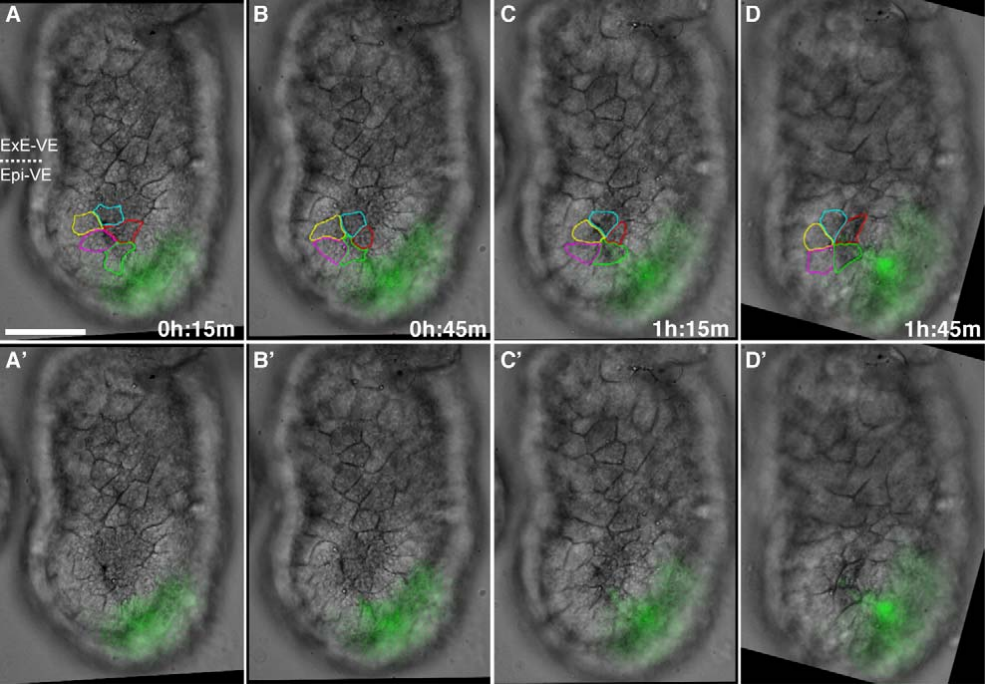
Rosettes form by cellular rearrangement. Time-lapse sequence of the VE surface of an embryo during AVE migration. The embryo is orientated with distal to the bottom and proximal to the top. The anterior of the embryo is to the right, marked by migrating AVE cells expressing Hex-GFP. Five focal planes were taken every 15 minutes and VE cell outlines determined on the basis of all focal planes. Selected images from the time-lapse sequence are shown, with (A–D) and without (A′–D′) cells outlined. A rosette can be seen to form in the Epi-VE by the rearrangement of non-neighbouring cells. The scale bar represents 50 µm. Also see Movies S2 and S3.

Rosette numbers increase during AVE migration (Figure 3A′), suggesting they are not static features of the VE. They are found predominantly in the Epi-VE, which is characterised by cell mixing [20]. To determine if rosettes in the mouse VE form by cell movement (as opposed, for example, to stereotypic patterns of cell division, or apoptosis of one cell drawing surrounding cells into a central point), we visualised cell outlines in the VE of cultured Hex-GFP embryos by DIC time-lapse microscopy. As before, we captured images from five focal planes at each time-point so cell outlines could be visualised unambiguously, and with a 15-minute time-lapse to achieve sufficient time-resolution to follow individual cells from one time-point to the next. Again, due to the strong curvature of the surface of the embryo, only a relatively small portion of the surface VE could be viewed in focus. In five embryos that remained in focus and in the field of view continuously for between 2 and 7 hours, we recorded a total of five rosettes forming—one rosette in each of three embryos and two rosettes in a fourth embryo. All these rosettes formed as a result of VE cells intercalating so that five or more cells met at a single central point to form a rosette. We did not observe apoptosis or cell division leading to rosette formation in any of these embryos. Cell tracking confirmed that in forming rosettes, cells that initially were not in contact with one another became neighbours (Figure 4 and Movies S2 and S3). Consistent with the distribution of rosettes in fixed embryos, we observed rosettes forming only in the Epi-VE.

We did not observe any rosettes resolving in our time-lapse recordings, suggesting that if they do resolve, it is on much longer time scales to their formation. We quantified rosettes in opacity renderings of ZO-1 stained 6.5 dpc embryos, approximately 20 hours after AVE migration, and found that while overall rosette density was significantly lower compared to “anterior” embryos, the average number of rosettes per embryo was significantly higher (Figure S4).

### Mathematical Modelling of AVE Migration

Our experimental observations show that AVE migration is accompanied by a decrease in mean polygon number in the Epi-VE and an increase in the number of rosettes. To explore possible roles for rosettes, we created a mathematical model that represents AVE migration within the mouse VE. A critical feature of the model is the ability to adjust the number of rosettes that form during migration simulations by changing a single parameter. We are thus able to observe how varying rosette numbers affects the emergent migration behaviour, whilst keeping all other parameters constant. Such computational experiments were intended to demonstrate whether rosettes are an important part of the migration process, or merely coincidental. We have recently described a 2-D version of such a model [36].

In our model, the apical surfaces of cells of the VE are represented by polygons lying on the surface of an ellipsoid. The polygonal representation is an abstraction of the cell shapes observed in vivo and captures key features such as edge- and neighbour-numbers. This framework is one of a class of cell-based models, including, for example, the cellular Potts model [37] and the cell-centre model [38]. Of these models, the vertex representation is the most appropriate in the context of AVE migration as it permits the explicit modelling of junctional rearrangements including rosette formation.

**Figure 5:**
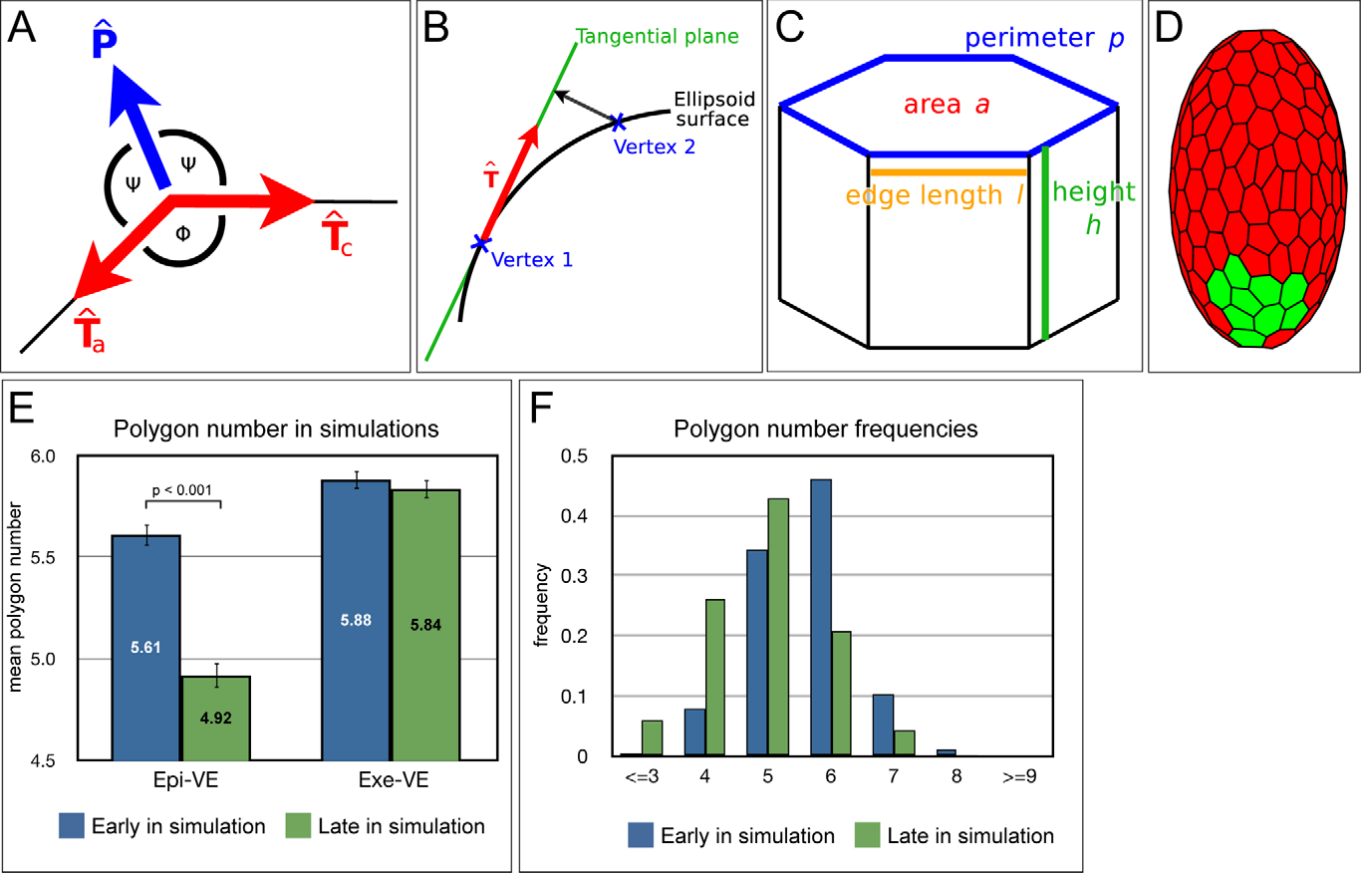
Modelling AVE migration. (A) Two-dimensional representation of force directions in the vertex model. At each vertex, tension forces act along the edges connecting neighbouring vertices, with unit direction vectors Tc (clockwise) and Ta (anti-clockwise). Pressure forces act normally at the vertex, bisecting the internal angle Φ, with unit direction vector P. (B) On the ellipsoid surface, forces act tangentially. To calculate the forces on a given vertex, its neighbours are projected onto the tangential plane. Unit direction vectors are then determined on this plane. (C) Each cell in the vertex model is 3-D, with associated height and volume. Forces act on the apical surface and depend on quantities such as surface area, edge lengths, height, and perimeter. (D) An initial cell configuration on the ellipsoid surface. Cells highlighted in green are the AVE. The polygon mesh represents the apical surfaces of cells of the VE. See Text S1 for further details. (E) Comparison of mean polygon number in the ExE-VE and Epi-VE early and late in simulation (roughly equivalent to “distal” and “anterior” embryos). As in wild-type embryos, there is a significant reduction in mean polygon number in the Epi-VE late in simulation as compared to early in simulation (Students *t* test, *p*<0.001). (F) Frequencies of polygon numbers early and late in simulations. Late in simulations, there is a significant difference in the distribution in the Epi-VE as compared to the ExE-VE, with an increase in four-sided cells and a decrease in six-sided cells (Kolmogorov-Smirnov test, *p*<0.001). There is no significant difference between the distribution in the ExE-VE and Epi-VE early in simulations. Early in simulations: *n* = 458 Epi-VE and 507 ExE-VE cells from five simulations. Late in simulations: *n* = 656 Epi-VE and 744 ExE-VE cells from five simulations.

The numerous forces acting on each cell in vivo are encapsulated by tension and pressure forces acting on the vertices of the polygonal cells. The directions in which these forces act in two-dimensions are shown in Figure 5A. To extrapolate to a three-dimensional ellipsoid, the forces act tangentially to the surface at each vertex (Figure 5B). Each cell also has a volume that is able to change over time. The cell's height along the apical-basal axis can be inferred by dividing the volume by apical surface area.

The equation for the tension force acting on a vertex due to one of the cells to which it belongs is given by:
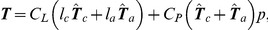
where *C_L_* and *C_P_* are constants, *l_c_* and *l_a_* are the lengths of the clockwise and anti-clockwise edges, respectively, and *p* is the length of the cell perimeter (Figure 5B,C).

The pressure force equation, meanwhile, is given by:
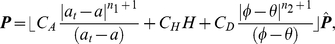
where *C_A_*, *C_H_*, and *C_D_* are constants, *a* is the cell area, *a_t_* is a target area, *H* is the height-to-area ratio, *θ* is the average internal angle of the cell (*θ* = *π*(*s*−2)/*s* for an *s*-sided polygon), 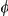 is the internal angle at the current vertex (see Figure 5A), and *n_1_*. and *n_2_* are integers.

We note that the exact form of the force equations does not affect the qualitative behaviour of our simulations. More information about the tension and pressure force equations can be found in Text S1.

By summing the contributions to the total force from each cell, an equation of motion for each vertex can be formulated. In this type of biological system, viscous forces dominate, and we therefore make the simplifying assumption that inertial forces can be neglected. The only additional parameter in the equations of motions is thus a viscosity parameter. The equation of motion for a vertex *i* is given by:
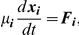
where *μ_i_* is the viscous coefficient, ***x*** *_i_* is the vertex position, and ***F*** *_i_* is the sum of all forces acting on the vertex.

The equations are solved iteratively, with vertices free to move anywhere in 3-D space. In vivo the cells of the VE adhere to the epiblast and extra-embryonic ectoderm below, maintaining the shape of the embryo. To simulate this restoring force, vertices are therefore projected back to the ellipsoid during each iteration (Figure 5D). The time-step in our simulations is kept sufficiently small so that this projection is small relative to the movement of the vertices.

In vivo, cells in the Epi-VE are highly labile relative to those of the ExE-VE [20]. To simulate this fact, we adjust the relative viscosity of the vertices in each half of the ellipsoid. A higher viscosity *μ* in the ExE-VE ensures that movement is more restricted in the proximal half of the embryo. In this way we are able to simulate the barrier to migration that occurs at the junction between the Epi-VE and ExE-VE.

Alongside the standard vertex movements driven by the forces described above, two types of junctional rearrangement have been observed experimentally, and are therefore included in the model. The first is a T1 transition, which has been used in many previous vertex models (e.g. Weliky and Oster [31], Farhadifar [32]). Secondly, an edge whose length falls below a certain threshold is allowed to contract to a single point, with the vertices at the ends of the edge joining together. Rosettes of various sizes occur when several neighbouring edges contract in succession. This is a key process in the model, allowing the effect of rosettes on migration to be investigated. The number of rosettes can be controlled by adjusting the threshold length at which vertices join together. Increasing the threshold leads to more rearrangements, while decreasing it leads to fewer rearrangements.

During AVE migration in vivo, cells grow in volume and proliferate, and the size of the embryo increases. In order for our model to be realistic it is important to include these processes. Each cell is assigned an initial volume, which grows logistically over time. Cell division is implemented stochastically, based on the ratio of cell volume to some target (see Text S1 for details). To simulate the concurrent increase in embryo size, the ellipsoid itself is allowed to grow over time. This requires an adjustment of the equations for the projection of vertices back to the ellipsoid surface (see Text S1 for details). The radius of the ellipsoid grows linearly, and over the course of migration increases by approximately 10%, in agreement with experimental observations.

We designate a subset of cells at the distal tip of our ellipsoid to be the AVE and induce them to migrate by adjusting the forces acting on their vertices (Figure 5D). This is achieved in practice by increasing the pressure force at one or more of the proximal-most vertices of each migrating cell. Increasing this force causes those vertices to move, which in turn affects the properties of the cell and results in the whole cell moving proximally. In reality migrating cells show protrusions in the direction of cell movement that can be several cell diameters long [13],[19]. Our migration force can therefore be thought of as the reaction of the main body of the cell to the directional cues provided by the protrusions.

### Rosettes Facilitate Coherent AVE Migration in Simulations of Migration

**Figure 6:**
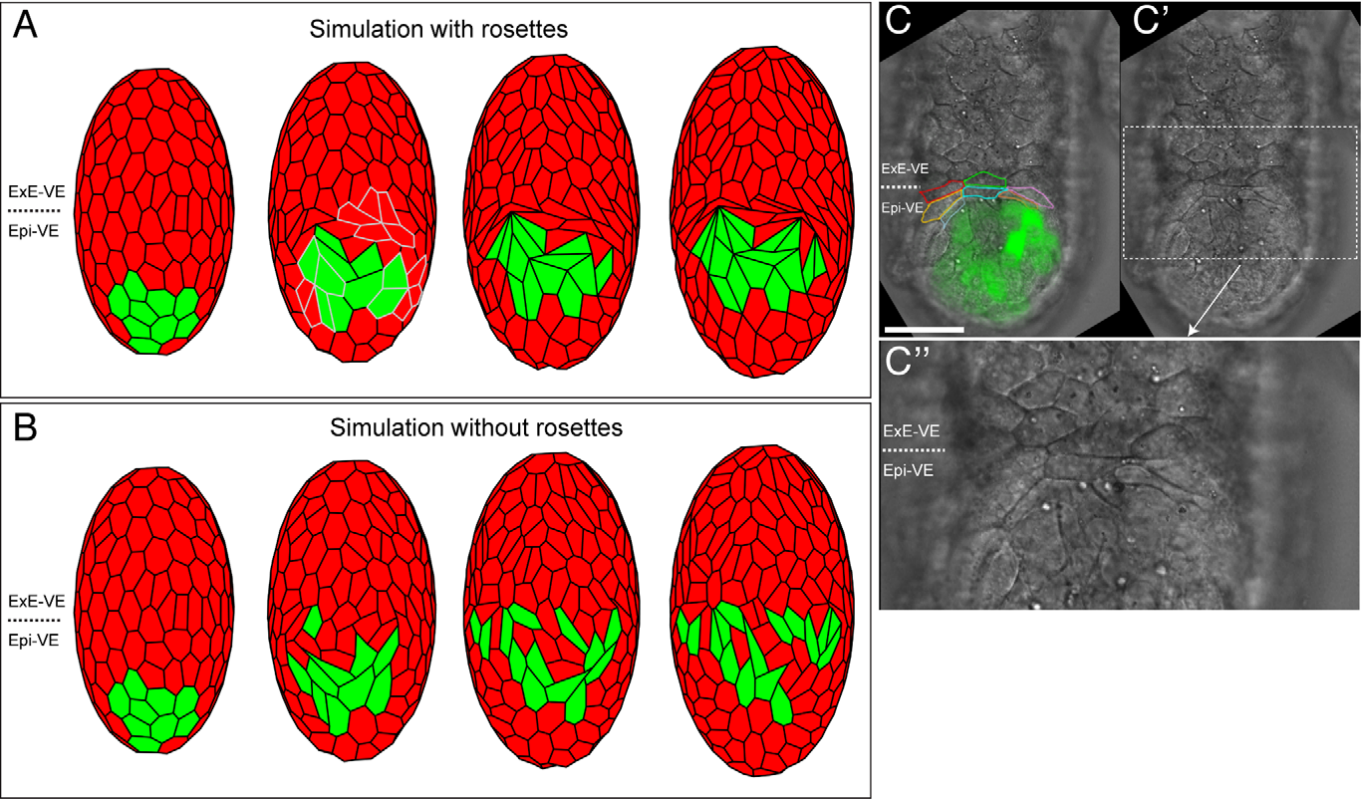
Simulation of AVE migration with and without rosettes. Cell migration simulations (A and B). Images taken at regular intervals, with initial configuration on the left and final distribution on the right. (A) High vertex-joining threshold. Cells migrate in a single group, when rosettes are allowed to form. Three rosettes are highlighted in grey in the second image from left. At the barrier between the epiblast and extra-embryonic ectoderm, cells form a crescent-shaped group very similar to that observed in experiments (C–C″). (B) Low vertex-joining threshold. The AVE breaks up in an abnormal manner, with cells dispersed. No crescent-shaped cells are visible at the boundary. (C) Frontal view of a 5.5 dpc embryo orientated with distal towards the bottom, proximal towards the top, and anterior facing the reader. Hex-GFP expressing AVE cells are in green. Cells at the barrier between the Epi-VE and ExE-VE (outlined in different colours) are elongated and form a crescent-shaped feature. (C′) Same embryo as in (C), without cells outlined or the GFP channel. (C″) High magnification view of the elongated cells at the boundary between the Epi-VE and ExE-VE. The scale bar represents 50 µm in (C) and (C′) and 25 µm in (C″). Also see Movie S4 and Text S1 for further details.

We initially simulated AVE migration with the vertex-joining threshold set at a level that allowed rosettes to form at a similar density to that observed experimentally. The AVE cells migrated in a manner similar to that seen in embryos, as an orderly, coherent group of cells. It was also found that cells ahead of the AVE were pushed against the ExE-VE forming a “crescent” shape very similar to that observed in embryos (Figure 6A,C and Movie S4).

To further test if our simulations were reasonable representations of experimental observations, we quantified polygon numbers both early and late in simulation (roughly equivalent to “distal” and “anterior” embryos, respectively).

As in cultured wild-type embryos, during simulations the Epi-VE underwent a significant reduction in mean polygon number (*p*<0.001, Student's *t* test) (Figure 5E). We also compared the frequency of different polygon numbers in the simulations. Similar to our observations in embryos, there was a significant shift in the frequencies of polygon numbers in the Epi-VE late in simulation as compared to early in simulations (*p*<0.001, Kolmogorov-Smirnov test) (Figure 5F), with a marked increase in the proportion of four-sided cells at the expense of six-sided cells.

Simulations were then run with a small vertex-joining threshold distance, thereby reducing the number of rosettes that form. All other parameters were kept constant. In this case AVE cells were able to migrate round the surface of the ellipsoid to the boundary with the ExE-VE, but in a dispersed manner not normally observed in embryos (Figure 6B and Movie S4). In these simulations the AVE breaks up into several clumps of cells with non-AVE cells between them, rather than maintaining its structure as a single coherent group.

The simulations suggest that the formation of rosette arrangements in the VE during AVE migration is required for the normal, *orderly* migration of AVE cells.

### Rosette Formation Requires PCP Signalling

**Figure 7:**
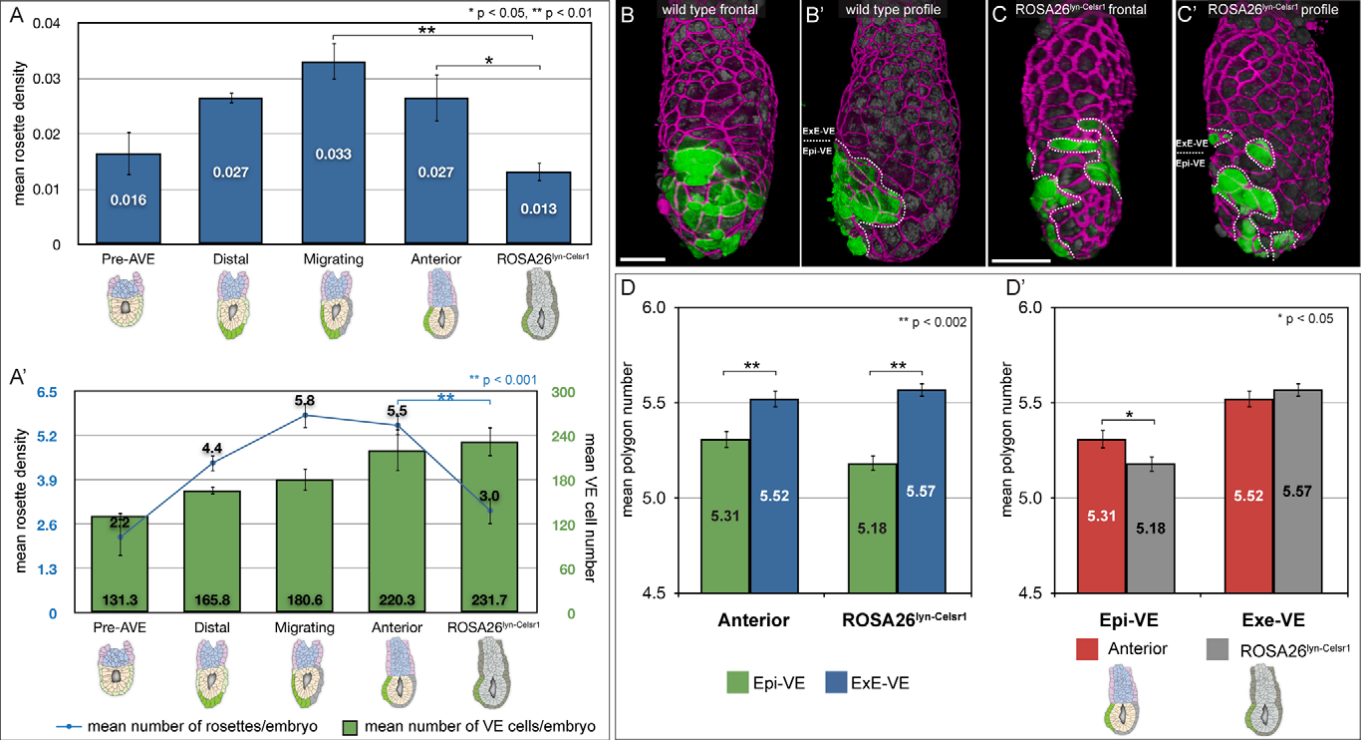
Abnormal AVE migration and cellular geometry in mutants with disrupted PCP signalling. (A) Rosette density (number of rosettes divided by total VE cell number) at different wild-type stages (“pre-AVE”: before AVE induction, *n* = 9; “distal”: AVE at distal tip before migration, *n* = 5; “migrating”: AVE migrating, *n* = 5; and “anterior”: AVE finished proximal migration and moving laterally, *n* = 4) and in *ROSA26^Lyn-Celsr1^* mutants (*n* = 7) with disrupted PCP signalling. There is a significant reduction in rosette density in *ROSA26^Lyn-Celsr1^* mutants compared with “migrating” and “anterior” embryos. (A′) The same data as in (A), but depicted as mean number of rosettes per embryo (blue line), and mean number of VE cells per embryo (green bars) at the various stages. *ROSA26^Lyn-Celsr1^* mutants have a comparable number of VE cells to stage matched “anterior” embryos, but show significantly fewer rosettes, leading to the reduced rosette density. (B, B′) En face and profile view of a representative “anterior” embryo, illustrating stereotypical ordered migration of AVE cells. The AVE is marked with a dotted line in (B′) and shows a single group of cells that does not extend more than half-way around the side of the embryo. (C, C′) En face and profile views of an equivalent stage *ROSA26^Lyn-Celsr1^* mutant, showing abnormal AVE migration. AVE cells appear to have broken into several groups (outlined with dotted lines in (C′)) and spread much more broadly within the Epi-VE and even into the ExE-VE. Cell outlines in the embryos in (B) and (C) were visualised by staining for ZO-1 (magenta), and AVE cells by the expression of Hex-GFP (green). Nuclei are visualised with DAPI (dim grey). (D) Comparison of mean polygon number in the Epi-VE and ExE-VE of “anterior” embryos (*n* = 480 Epi-VE and 409 ExE-VE cells from three embryos) and equivalent stage *ROSA26^Lyn-Celsr1^* mutants (*n* = 563 Epi-VE and 546 ExE-VE cells from four embryos). As in wild-type “anterior” embryos, the mean polygon number in the Epi-VE of *ROSA26^Lyn-Celsr1^* mutants is significantly lower than that in the ExE-VE. (D′) The same polygon number data grouped according to the VE region. Though the mean polygon number in the ExE-VE is comparable for “anterior” and *ROSA26^Lyn-Celsr1^* embryos, in the Epi-VE it is significantly lower in *ROSA26^Lyn-Celsr1^* embryos, suggestive of increased disequilibrium in cell packing. The scale bar represents 50 µm. *p* values shown on the graphs were determined using Student's *t* test.

PCP signalling coordinates cell polarisation and rearrangement across fields of cells in a variety of contexts. PCP signalling is disrupted in the *ROSA26^Lyn-Celsr1^* mouse line [20]. To determine if rosette formation is perturbed in these mutants, we quantified them in mutant embryos dissected at 5.75 dpc, a stage comparable to the wild-type “anterior” group. Mutants had a significantly reduced rosette density when compared to “anterior” embryos (*p*<0.05, Student's *t* test) (Figure 7A). *ROSA26^Lyn-Celsr1^* embryos are similar in size to “anterior” embryos and the reduction in rosette density is the result of a significant reduction in the average number of rosettes per embryo (*p*<0.001, Student's *t* test) (Figure 7A′).

The AVE migrates in *ROSA26^Lyn-Celsr1^* mutants, but in the majority of cases (six out of eight embryos) was abnormally dispersed, in a manner reminiscent of simulations in our model when rosettes were not allowed to form (Figures 7B–C and 6B). These mutants also show a variety of other AVE migration abnormalities such as unilateral whorls or migration into the ExE-VE [20].

We determined the polygon numbers of VE cells in *ROSA26^Lyn-Celsr1^*embryos. As with wild-type “anterior” embryos, the mean polygon number was significantly lower in the Epi-VE compared to the ExE-VE (*p*<0.001, Student's *t* test) (Figure 7D). Similarly, the frequency of polygon numbers in the ExE-VE and Epi-VE was found to be significantly different (*p*≤0.001, Kolmogorov-Smirnov test) (Figure S1G).

Interestingly, when compared to the Epi-VE of wild-type “anterior” embryos, the polygon number in the Epi-VE of *ROSA26^Lyn-Celsr1^* embryos was significantly lower (*p*<0.05, Student's *t* test) (Figure 7D), suggesting that there was increased disequilibrium in Epi-VE cell packing in the absence of rosettes.

## Discussion

### Increase in Cell Packing Disequilibrium during AVE Migration

Prior to AVE migration, the distribution of cell polygon number is comparable in the Epi-VE and ExE-VE, with a peak between five and six sides. This distribution is different from the equilibrium distribution reported by Gibson et al. for a variety of metazoan epithelia that have a distinct peak at six-sided cells [28]. One possible explanation for this difference is that while the epithelia considered by Gibson et al. are all relatively flat (Drosophila wing imaginal disc, Xenopus tail epidermis, and Hydra external epidermis), the mouse VE is very highly curved with an average of fewer than 20 cells around a circumference of about 300 microns. This is likely to impose different constraints on the packing of cells in the VE when compared to other epithelia.

During AVE migration stages, mean polygon number drops and polygon distribution shifts towards three- and four-sided cells, but only in the Epi-VE (Figure 1B, B′, and Figure S2H). The ExE-VE in contrast does not show so marked a reduction in mean polygon number. This is consistent with time-lapse data which show that the Epi-VE and ExE-VE are distinct in their behaviour, the former undergoing a great deal of cell mixing with cells continuously changing shape, while the latter is relatively static [20]. A specific link between AVE migration and changes in epithelial topology is reinforced by *Nodal^Δ600/lacZ^* and *Cripto* ^−/−^ embryos in which the AVE fails to migrate and in which the mean polygon number in the Epi-VE remains close to that in wild-type embryos in which the AVE has not yet started migrating (Figure 1B and B′).

A reduction in mean polygon number is also observed in the Epi-VE of cultured embryos, where the same set of VE cells is monitored during AVE migration. This indicates that the reduction in mean polygon number is due at least in part to dynamic changes in the packing of existing VE cells taking place on the time scale of 4 hours rather than, for example, new cells with fewer cell edges arising through division. Again, the change in polygon number is restricted to Epi-VE cells, consistent with this being the region that is behaviourally labile and to which AVE cell migration is restricted [20]. These findings suggest that during AVE migration the Epi-VE is in a state of increased disequilibrium with respect to cell packing.

### Rosettes Aid in the Orderly Migration of the AVE

We observe multi-cellular rosettes in the Epi-VE, a striking conformation of cells that deviates greatly from the hexagonal packing considered to be the equilibrium arrangement of cells in epithelia. In the Drosophila germband, rosettes have been shown to be transient intermediaries of the long-range coordinated cell movements of convergent-extension [25]. There are, however, no convergent-extension movements in the mouse VE and rosettes appear to play a different role in this context.

The significant increase in rosettes during AVE migration in wild-type embryos and the reduction in rosettes in mutants with a failure of AVE migration point to a specific role for rosettes in AVE migration (Figure 3A). This is further supported by the observation that rosettes are predominantly found in the Epi-VE, the region of the VE to which AVE migration is restricted. However, rosettes are not restricted to the anterior region of the Epi-VE but more or less evenly distributed throughout the Epi-VE (Figure 3B), with only a minority of rosettes (8%) including any Hex-GFP positive AVE cells. This suggests that rosettes are not involved specifically in driving AVE cell movement or determining the direction in which they migrate, but play a more general role in the Epi-VE during AVE migration.

Our mathematical model predicts that rosettes are essential for *ordered* migration, in which the AVE cells migrate as a coherent group. When simulations are run with fewer rosettes, AVE migration still takes place, but in an abnormally dispersed manner. It is only when rosettes are allowed to form that AVE migration is much more orderly and closely resembles that seen in actual embryos. This is confirmed by experiments using *ROSA26^Lyn-Celsr1^* mutant embryos in which PCP signalling is disrupted [20] and significantly fewer rosettes are formed. Such embryos exhibit AVE migration but in an abnormally disordered fashion. Rosettes in the mouse VE are therefore not essential to drive AVE migration (in the sense they are understood to contribute to convergent extension in the Drosophila germ band), but appear to have the subtler role of modulating AVE migration so that it occurs in a stereotypic, orderly manner.

AVE cells have been shown to migrate in response to a directional cue from Dkk1 [17]. AVE cells migrate within an intact epithelial sheet by cell intercalation [19],[20]. It is not only AVE cells that show this intercalatory behaviour, but also other surrounding cells in the Epi-VE [20]. This suggests that intercalation among AVE and non-AVE cells in the Epi-VE needs to be coordinated, to allow AVE cells to “negotiate” their way through a field of Epi-VE cells to arrive at the prospective anterior. Our time-lapse experiments show that rosettes form as a result of cell intercalation and that the majority of cells participating in rosettes, though in the Epi-VE, are not AVE cells. PCP signalling is active in the Epi-VE and influences AVE migration [20]. When PCP signalling is disrupted, there are significantly fewer rosettes though the AVE still migrates (albeit abnormally), suggesting that rosette formation is not a passive response to AVE migration but is actively dependent on PCP signalling.

We interpret these results to suggest the following working model of AVE migration. Though AVE cells migrate in response to an extrinsic guidance cue, since they have to migrate through an intact epithelium, this movement has to be achieved through cell intercalation that has to be coordinated between the migrating AVE cells and surrounding non-AVE cells. We suggest that the role of PCP signalling in the Epi-VE is to coordinate this intercalation, at least in part via the formation of rosettes. We suggest rosettes facilitate orderly AVE migration by buffering the increased disequilibrium in cell packing in the Epi-VE accompanying the directional movement of AVE cells. Consistent with this view, after AVE migration the mean polygon number in embryos with disrupted PCP signalling is significantly lower than that in the Epi-VE of equivalent stage wild-type embryos, indicative of increased epithelial disequilibrium in the absence of rosettes.

How might rosette formation buffer the disequilibrium of cell packing in the Epi-VE? One possibility is that it allows non-AVE cells to group together and behave as a single unit, which in some way makes it easier for AVE cells to migrate through the VE epithelium. Though we observe several rosettes forming in time-lapse experiments, we do not observe any rosettes resolving. This suggests either that once formed they are relatively static features or that they resolve over different time-scales than those over which they form. Rosette density in 6.5 dpc embryos (approximately 20 hours after AVE migration) is significantly lower than that in “anterior” embryos, but this is due to the significant increase in size of embryos between these two stages rather than to a reduction in the number of rosettes. A total of 6.5 dpc embryos have a significantly higher average number of rosettes per embryo as compared to “anterior” embryos (Figure S4), consistent with the notion that rosettes formed during AVE migration might accumulate over time rather than resolve. A detailed study of the dynamics of rosettes will help address how precisely rosettes aid in the orderly migration of AVE cells, the mechanistic basis for their formation, and clarify whether they resolve. Recent developments in high resolution, low photo-damaging imaging technology such as light sheet microscopy [39],[40] now make it feasible to monitor cell movements on the surface of the cylindrical embryo over extended time-scales and will help resolve these issues.

### Modelling Cell Movements in Epithelia

In contrast to convergent-extension movements where all cells undergo a coordinated medio-lateral intercalation leading to tissue elongation, during AVE migration a subset of cells migrates directionally within a larger field of cells that undergoes cell rearrangement without extensive changes to the overall shape of the epithelium. Since the VE is arranged as a cylinder, it provides an appropriate model for the study of cell movements in other epithelia on elongated curved surfaces, such as lung buds, ureteric buds, or developing intestinal villi.

Our mathematical model of cell movements in the VE, in combination with experimental intervention, provides a powerful tool for the study of directed cell movements within epithelia. It is built on simple assumptions, incorporating forces acting upon cells, cell division, directional movement of a subset of cells, a behavioural “barrier” to migration, and the ability of cells to rearrange to form rosettes. Although the cells in our model have volume and height, they are not fully 3-D, in the sense that forces act only on apical surfaces, and there is no consideration of the fact that neighbouring cells might be at different heights. As further biological data are obtained, 3-D vertex models such as that of Honda et al. [41] may become desirable in exploring the cellular dynamics of epithelia such as the VE. However, representing the tissue as a 2-D sheet as we have currently done has proved informative in exploring the role of rosettes. From just the starting conditions of our model, behaviour emerges in simulations similar to that observed experimentally—for example, the formation of a “crescent” where cells ahead of the AVE are pushed against the ExE-VE, the reduction in mean polygon number during migration, and the abnormally broad and disordered migration of AVE cells when rosettes are not allowed to form. This emergent behaviour reinforces the potential of our model as a tool in probing cell migration in the VE and other epithelia.

## Materials and Methods

### Mouse Strains, Husbandry, and Embryo Collection

Genetically modified mice were maintained on a mixed C57Bl/6 CBA/J background. The *Hex-GFP* line was bred into the various mutant backgrounds to enable the AVE to be followed. Embryos carrying the *Hex-GFP* transgene were obtained by crossing homozygous *Hex-GFP* studs with CD1 females (Charles River). All mice were maintained on a 12 hour light, 12 hour dark cycle. Noon on the day of finding a vaginal plug was designated 0.5 dpc. Embryos of the appropriate stage were dissected in M2 medium (Sigma) with fine forceps and tungsten needles.

### Immunohistochemistry

Secondary only controls were done to verify the specificity of secondary antibodies. Embryos were fixed in 4% PFA in PBS at 4°C for 30 minutes; washed at room-temperature thrice for 5 minutes each in 0.1% Triton-X100 in PBS (PBT); incubated in 0.25% Triton-X100 in PBS for 15 minutes; washed thrice in PBT; blocked with 2.5% donkey serum, 2.5% goat serum, and 3% Bovine Serum Albumin (BSA) in PBT for 1 hour; incubated overnight at 4°C in primary antibodies diluted in 100 µl PBT; washed five times in PBT for 5 minutes each, with a final additional wash for 20 minutes; incubated at room temperature in the appropriate secondary diluted in 100 µl PBT for 2 hours or overnight; washed in PBT five times for 5 minutes and once for 15 minutes; and finally mounted with Vectashield mounting media containing DAPI (Vector Labs H-1200). Antibodies used were: Rabbit anti-ZO-1 (Zymed laboratories 61-7300) 1∶100 and Alexa Fluor 555 donkey anti-rabbit IgG (Invitrogen A-31572).

### Confocal Microscopy and Volume Rendering

Fixed samples were imaged on Zeiss LSM 510META and Zeiss LSM 710 confocal microscopes using 20×/0.75NA or 40×/1.2NA lenses as appropriate. DAPI was excited at 405 nm, EGFP at 488 nm, and Alexa Fluor 555 at 543 nm. Z-stacks of entire embryos were acquired at a 0.8 µm interval using non-saturating scan parameters. Z-stacks of embryos were opacity rendered as 3-D volumes using Volocity Software (Improvision, UK). Figures were prepared with Adobe CS2 Photoshop and Illustrator (Adobe Inc).

### Polygon Number Quantitation and Statistical Analysis

Opacity rendered views of embryos were rotated through 360°, printed out, and the polygon number of each cell determined manually as the number of neighbours it had. Each cell was given a unique reference number to avoid being counted twice. Data were tabulated in Microsoft Excel and Apple Numbers 2009. Statistical analysis was performed using SPSS Statistics 17.0 and Apple Numbers 2009.

### Time-Lapse Microscopy

Culture media consisted of 50% home-made heat-inactivated mouse serum and 50% CMRL (Invitrogen) supplemented with L-glutamine, equilibrated at 37°C and 5% CO2 for at least 2 hours prior to imaging. Embryos were transferred into the pre-equilibrated media in Lab-TekII Coverglass bottomed eight-well rectangular chambers (Nalge Nunc International) and imaged for up to 8 hours on an inverted Zeiss 710 confocal microscope equipped with an environmental chamber to maintain conditions of 37°C and 5% CO_2_. Embryos were imaged with a water immersion 40×/1.2 NA objective every 15 minutes. At every time point, a Z-stack of five focal planes separated by 10.78 µm was captured. EGFP marking AVE cells was excited at 488 nm and DIC images were acquired with the confocal's transmitted light PMT.

### Embryo Genotyping

Antibody stained confocal imaged embryos were recovered from slides; washed in syringe filtered PBT thrice for 5 minutes; washed in lysis buffer (50 mM Tris HCl pH 8–8.5, 1 mM EDTA, 0.5% Tween-20) for 5 minutes; transferred into PCR strips containing lysis buffer (16 µl for 5.5 dpc embryos) and Proteinase K (1 µl 20 mg/ml PK per 25 µl of embryo lysis buffer); lysed at 55°C for 1 hour; and the Proteinase K inactivated by incubating at 95°C for 10 minutes. PCR genotyping was performed using 3 µl of lysed embryo as template, the appropriate primers, and Illustra PuReTaq Ready-To-Go PCR Beads (GE Healthcare Catalogue No. 27-9557-01 (0.2 ml tubes/plate 96)). Cripto mutants were identified by their failure of AVE migration phenotype.

### PCR Primers and Conditions

Primers for *ROSA26^Lyn-Celsr1^*: R1: 5′AAAGTCGCTCTGAGTTGTTAT3′; R2: 5′GCGAAGAGTTTGTCCTCAACC3′; R3: 5′GGAGCGGGAGAAATGGATATG3′. Bands expected: 250 bp mutant (R1+R2) and 500 bp (R1+R3).

Primers for *Nodal^lacZ^*: LacZ-5: 5′CCGCGCTGTACTGGAGGCTGAAG3′; LacZ-3: 5′ATACTGCACCGGGCGGGAAGGAT3′; A: 5′ATGTGGACGTGACCGGACAGAACT3′; B: 5′CTGGATGTAGGCATGGTTGGTAGGAT3′. Bands expected: 750 bp mutant and 700 bp.

Primers for Nodal^Δ600^: Δ600-5: 5′GCTAGTGGCGCGATCGGAATGGA3′; Δ600-6: 5′AAGGGAAGTGAACTGGAAAGGTATGT3′. Bands expected: 350 bp mutant and 950 bp.

## Supporting Information

Figure S1 Comparison of polygon frequencies in Epi-VE and ExE-VE. There is a significant difference between the Epi-VE and ExE-VE in the distribution of polygon numbers in “migrating” and “anterior” embryos. This difference is not seen in the AVE arrest mutants *Nodal^Δ600/lacZ^* and *Cripto* ^−/−^.
(PDF)

Figure S2 Comparison of polygon frequencies in Epi-VE of different types of embryos. There is a significant difference in polygon frequencies in the Epi-VE of “migrating” and “anterior” embryos as compared to “distal” embryos. This difference is not seen in the AVE arrest mutants *Nodal^Δ600/lacZ^* and *Cripto* ^−/−^.
(PDF)

Figure S3 *Nodal^Δ600/lacZ^* and *Cripto* ^−/−^ embryos are similar to wild-type embryos in shape. Representative opacity renderings of two wild-type (A, A′), two *Nodal^Δ600/lacZ^* (B, B′), and two *Cripto* ^−/−^ (C, C′) embryos showing that they are similar in shape. Cell outlines are visualised by staining for the apical junction marker ZO-1. The scale bar represents 50 µm.
(PDF)

Figure S4 Rosettes in 6.5 dpc embryos. (A) Rosette density (number of rosettes divided by total VE cell number) at 5.75 dpc (“anterior”: AVE finished proximal migration and moving laterally, *n* = 4) and 6.5 dpc (*n* = 7). There is a significant reduction in rosette density in 6.5 dpc embryos. (A′) The same data as in (A), but depicted as mean number of rosettes per embryo (blue line), and mean number of VE cells per embryo (green bars) at the two stages. The 6.5 dpc embryos have approximately double the number of rosettes as “anterior” embryos, but 4-fold more VE cells, leading to an overall reduction in rosette density.
(PDF)

Text S1 Details of mathematical modelling of AVE migration.
(PDF)

Movie S1 Rosettes are composed of a single layer of cells all in direct contact with the epiblast. The animation shows an embryo in which the epiblast and ExE have been segmented in grey and individual cells of a rosette in the VE have been segmented in different colours. This allows one to make the epiblast “transparent” and examine the basal surface of the rosette to confirm that all the cells of the rosette contact the epiblast.
(MOV)

Movie S2 Rosettes form by cellular rearrangement. Time-lapse movie of rosettes forming in the VE during AVE migration. Representative cells are outlined in different colours so they can be easily followed. AVE cells are marked by Hex-GFP fluorescence (green). Rosettes can be seen forming in the Epi-VE by the rearrangement of non-neighbouring cells. The time interval between frames is 15 minutes. The scale bar represents 50 µm.
(MOV)

Movie S3 Rosettes form by cellular rearrangement. Time-lapse movie of another example of rosettes forming in the VE during AVE migration. Representative cells are outlined in different colours so they can be easily followed. AVE cells are marked by Hex-GFP fluorescence (green). Rosettes can be seen forming in the Epi-VE by the rearrangement of non-neighbouring cells. The time interval between frames is 15 minutes. The scale bar represents 50 µm.
(MOV)

Movie S4 Simulations of AVE migration in the presence and absence of rosettes. Simulations were run with a large threshold distance at which vertices join (allowing rosettes to form—at left) and with a very small threshold distance at which vertices join (preventing many rosettes from forming—at right). All other parameters were kept exactly the same. For simulations in which rosettes can easily form, AVE cells migrate as a group in a manner very similar to that observed in cultured embryos. In simulations with reduced rosette formation, AVE cells migrate in an abnormal manner, splitting into separate groups that spread more broadly than normally observed.
(MOV)

## Abbreviations

AVE: anterior visceral endoderm
dpc: days *post coitum*
Dvl-2: dishevelled-2
Epi-VE: visceral endoderm overlying the epiblast
ExE: extra-embryonic ectoderm
ExE-VE: visceral endoderm overlying the extra-embryonic ectoderm
PCP: planar cell polarity
VE: visceral endoderm
