# Supplementary material for: Multi-Cellular Rosettes in the Mouse Visceral Endoderm Facilitate the Ordered Migration of Anterior Visceral Endoderm Cells
Source: PLoS Biol. 2012 Feb 7;10(2):e1001256. doi: 10.1371/journal.pbio.1001256 (PMC3274502; doi:10.1371/journal.pbio.1001256)

Supplementary figure S1

Kolmogorov-Smirnov pair-wise comparison of frequencies of polygon numbers between the Epi-VE and ExE-VE of different types of embryos.

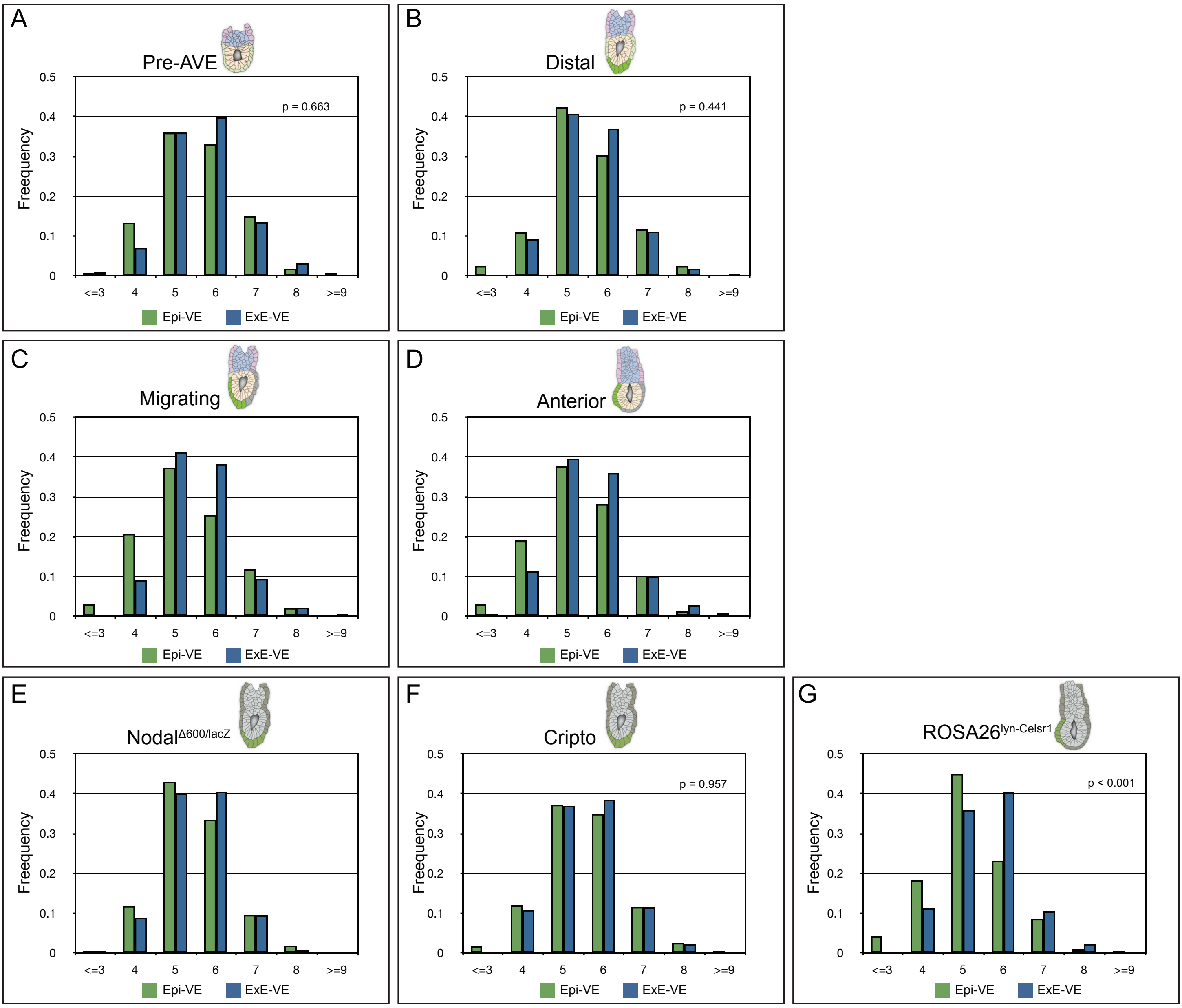

Supplement: Figure S1 — Comparison of polygon frequencies in Epi-VE and ExE-VE. There is a significant difference between the Epi-VE and ExE-VE in the distribution of polygon numbers in “migrating” and “anterior” embryos. This difference is not seen in the AVE arrest mutants NodalΔ600/lacZ and Cripto −/−. (PDF) [file pbio.1001256.s001.pdf]
