# Supplementary material for: Multi-Cellular Rosettes in the Mouse Visceral Endoderm Facilitate the Ordered Migration of Anterior Visceral Endoderm Cells
Source: PLoS Biol. 2012 Feb 7;10(2):e1001256. doi: 10.1371/journal.pbio.1001256 (PMC3274502; doi:10.1371/journal.pbio.1001256)

Supplementary figure S2

Kolmogorov-Smirnov pair-wise comparison of frequencies of polygon numbers between the ***Epi-VE*** of different types of embryos.

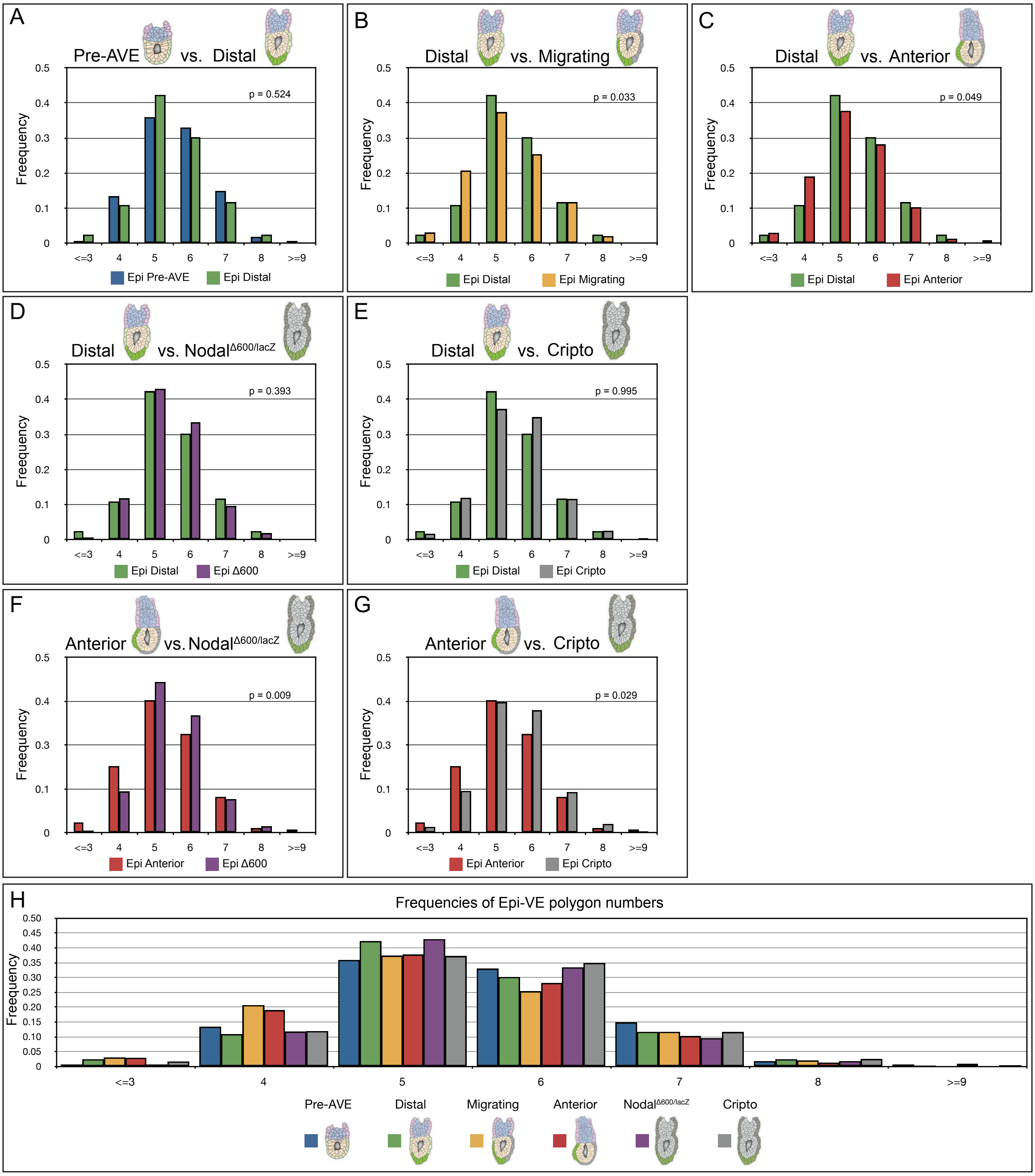

Supplement: Figure S2 — Comparison of polygon frequencies in Epi-VE of different types of embryos. There is a significant difference in polygon frequencies in the Epi-VE of “migrating” and “anterior” embryos as compared to “distal” embryos. This difference is not seen in the AVE arrest mutants NodalΔ600/lacZ and Cripto −/−. (PDF) [file pbio.1001256.s002.pdf]
