# Supplementary figures and images for: Multi-Cellular Rosettes in the Mouse Visceral Endoderm Facilitate the Ordered Migration of Anterior Visceral Endoderm Cells
Source: PLoS Biol. 2012 Feb 7;10(2):e1001256. doi: 10.1371/journal.pbio.1001256 (PMC3274502; doi:10.1371/journal.pbio.1001256)

Supplementary figure S3

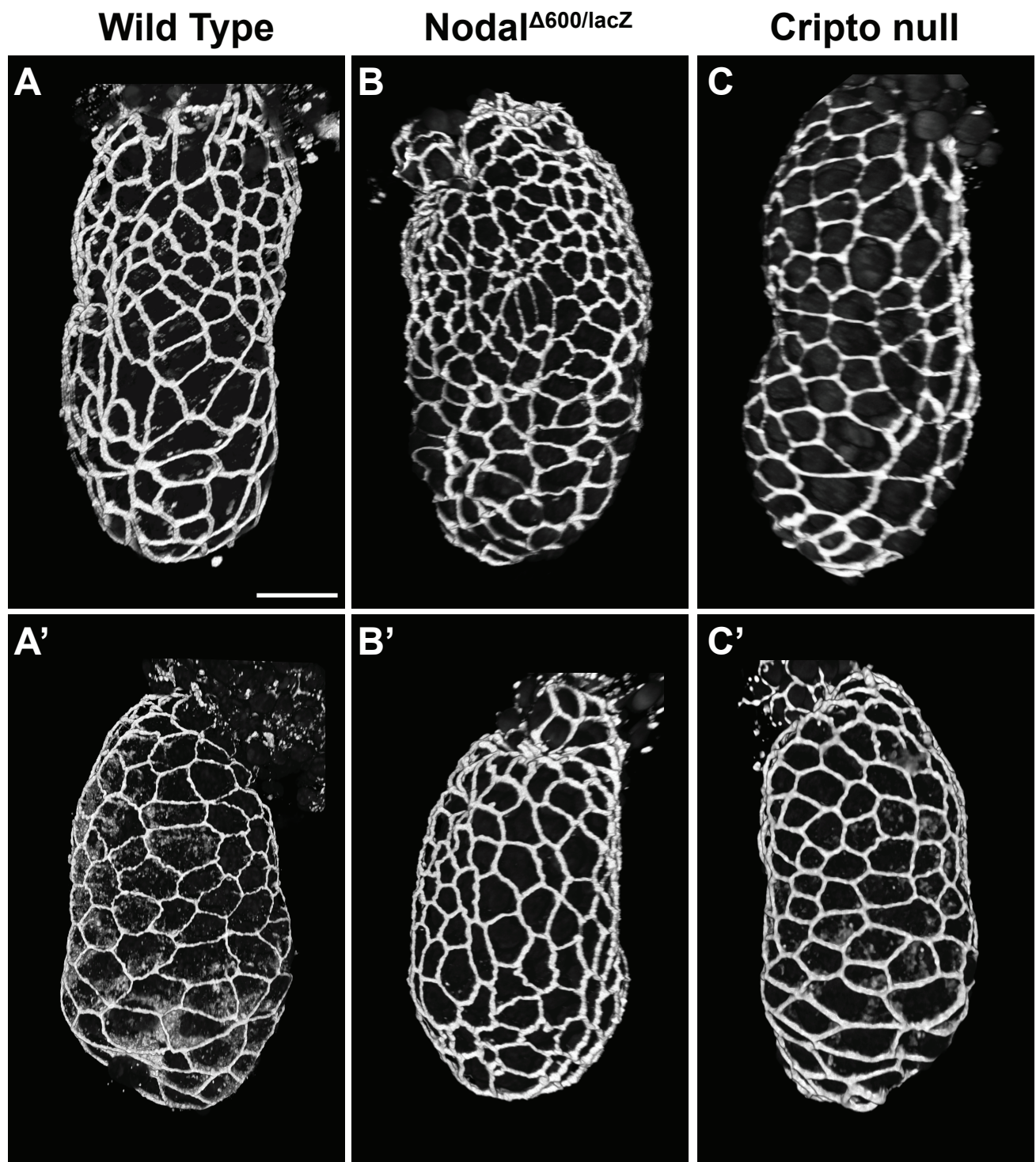

Supplement: Figure S3 — NodalΔ600/lacZ and Cripto −/− embryos are similar to wild-type embryos in shape. Representative opacity renderings of two wild-type (A, A′), two NodalΔ600/lacZ (B, B′), and two Cripto −/− (C, C′) embryos showing that they are similar in shape. Cell outlines are visualised by staining for the apical junction marker ZO-1. The scale bar represents 50 µm. (PDF) [file pbio.1001256.s003.pdf]

Supplementary Figure S4

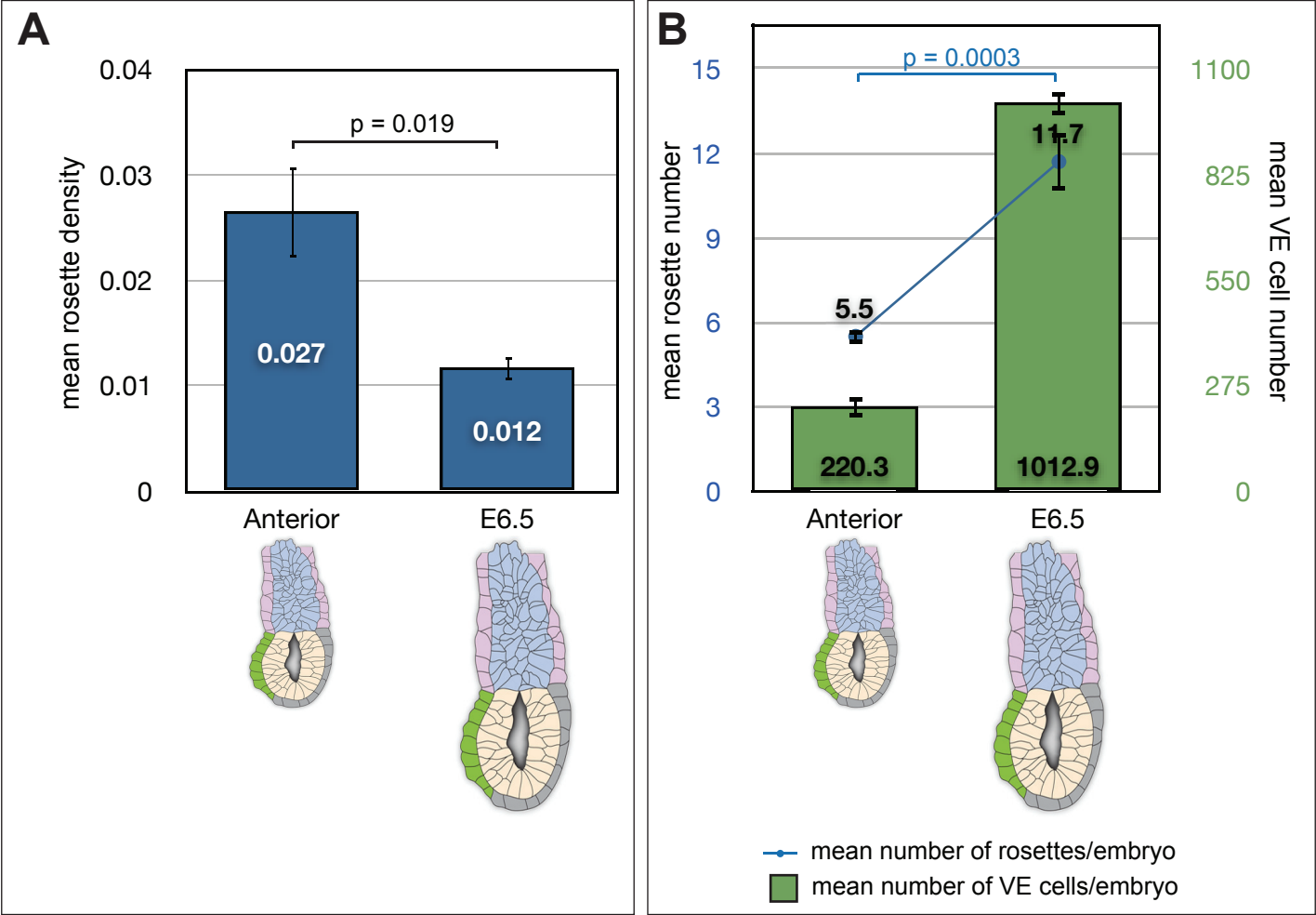

Supplement: Figure S4 — Rosettes in 6.5 dpc embryos. (A) Rosette density (number of rosettes divided by total VE cell number) at 5.75 dpc (“anterior”: AVE finished proximal migration and moving laterally, n = 4) and 6.5 dpc (n = 7). There is a significant reduction in rosette density in 6.5 dpc embryos. (A′) The same data as in (A), but depicted as mean number of rosettes per embryo (blue line), and mean number of VE cells per embryo (green bars) at the two stages. The 6.5 dpc embryos have approximately double the number of rosettes as “anterior” embryos, but 4-fold more VE cells, leading to an overall reduction in rosette density. (PDF) [file pbio.1001256.s004.pdf]
