# Supplementary material for: Multi-Cellular Rosettes in the Mouse Visceral Endoderm Facilitate the Ordered Migration of Anterior Visceral Endoderm Cells
Source: PLoS Biol. 2012 Feb 7;10(2):e1001256. doi: 10.1371/journal.pbio.1001256 (PMC3274502; doi:10.1371/journal.pbio.1001256)
Supplement: Text S1 — Details of mathematical modelling of AVE migration. (PDF) [file pbio.1001256.s005.pdf]

Multi-cellular rosettes in the mouse visceral endoderm facilitate the ordered migration of AVE cells

Georgios Trichas, Aaron Smith, Natalia White, Vivienne Wilkins, Abigail Moore, Bradley Joyce, Jacintha Sugnaseelan, Tristan Rodriguez, David Kay, Ruth Baker, Philip K. Maini & Shankar Srinivas.

Supplementary Information

Contents

1 Introduction **2**

2 Creating an initial configuration **2**

3 Force laws **3**

4 Equations of motion **6**

5 Constraining vertices to the ellipsoid surface **7**

6 Junctional rearrangements **8**

7 Cell growth and proliferation **11**

8 Simulating migration **12**

9 Modelling the barrier **12**

1 Introduction

This document describes a vertex model used to simulate AVE migration in the mouse embryo. The model draws on previous formulations, such as those of Weliky and Oster (1990); Farhadifar et al. (2007); Rauzi et al. (2008); Landsberg et al. (2009); Aegerter-Wilmsen et al. (2010). A two-dimensional (2D) version of this model is described in Smith et al. (2011). These models are extended by implementation on an ellipsoid surface, in order to represent a realistic geometry for the mouse embryo. A new type of junctional rearrangement is included, allowing close vertices to join together, and facilitating the formation of rosettes. A migratory force is introduced to allow the effects of migrating cells on the system to be examined, as well as a method for modelling the barrier between the epiblast and extra-embryonic endoderm. The model aims to capture the essential features of the migratory process, and subsequently test how they change when the ability of rosettes to form is disrupted. Simulations are robust to slight changes in the form of the force equations, and a range of parameter-value combinations lead to the observed emergent behaviour.

2 Creating an initial configuration

The icosahedron is a regular polyhedron composed of twenty equilateral triangles, which meet at twelve nodes (Fig. 1(a)). Node positions can be chosen to lie on the unit sphere. This coarse triangular mesh can be refined by subdividing each triangle into four smaller triangles. The mid-points between neighbouring nodes are found and used to create four new triangles inside each current one. The new nodes are then projected back onto the unit sphere. This process can be repeated *ad infinitum* to create as many triangles as required. Fig. 1(b) shows the resultant mesh after two complete iterations, in other words each triangle of the icosahedron is now 16 smaller triangles.

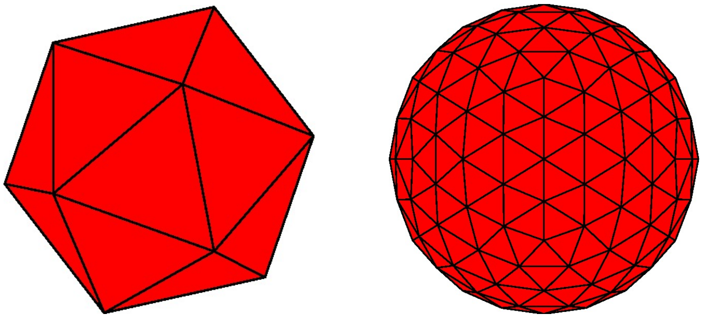

(a) (b)

Figure 1: *(a) An icosahedron, with 20 triangular faces meeting at 12 vertices that lie on the unit sphere. (b) The icosahedron is refined twice, so that each triangular face becomes 16 smaller triangles, yielding 320 triangles meeting at 162 vertices.*

This series of triangles is then transformed into a cell configuration, by taking the dual of the triangular mesh. Each node of the triangular mesh is used as the centre of a cell, and the centre of each triangle is a vertex in the cell mesh. The number of cells is therefore equal to the number of nodes in the triangular mesh. Fig. 2(a) shows the cell mesh dual of the triangular mesh from Fig. 1(b). The cell mesh can then finally be stretched by a factor c along one axis to create an ellipsoidal surface. The equation for the resultant ellipsoid is given by

$$x^2 + y^2 + \frac{z^2}{c^2} = 1.$$

Adding some random noise to the vertex positions gives a less regular starting condition (Fig. 2(b)).

3 Force laws

We make the simplifying assumption that the numerous forces acting on a cell *in vivo* can be reduced to a few simple components, which each contribute to one of two types of force acting on the vertices of each cell. These net forces are the sum of their respective components, and act in the directions shown

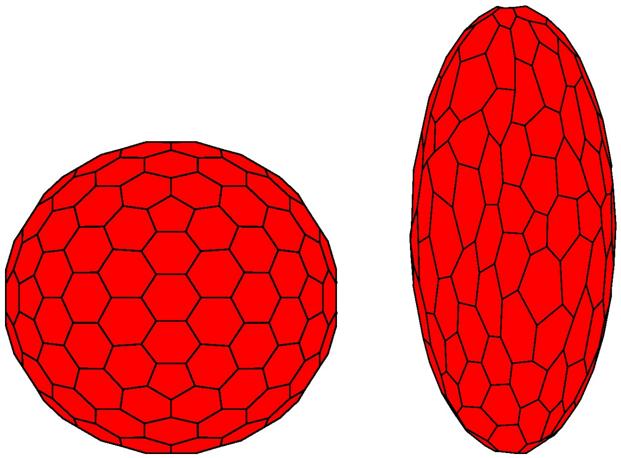

Figure 2: *(a) Taking the dual of the triangular mesh, a cell mesh on the unit sphere is obtained. (b) By stretching the cell mesh and adding some random noise to the vertex positions we obtain a cell mesh on the ellipsoid.*

in Fig. 3(a). Forces that act along the edges connecting vertices are labelled 'tension' forces, with dimensionless unit direction vectors $\hat{\mathbf{T}}_c$ (clockwise) and $\hat{\mathbf{T}}_a$ (anti-clockwise). Those forces that act in an outwards direction, bisecting the angle ϕ between adjacent edges, are 'pressure' forces, with unit direction vector $\hat{\mathbf{P}}$. Fig. 3(b) shows a single cell, with key parameters labelled. The cell mesh represents the top (apical) surface of each cell, and forces act exclusively on this level.

The tension force on each vertex consists of two components, which together represent cell-cell adhesion and contractility of the actin-myosin ring. The first component depends on the length of the two edges connecting the vertex to its clockwise and anti-clockwise neighbours, and is a line tension. The second component, meanwhile, depends on the length of cell perimeter. The tension force contribution due to a cell on a vertex is given by

$$\mathbf{T} = C_L\left(l_c\hat{\mathbf{T}}_c + l_a\hat{\mathbf{T}}_a\right) + C_P\left(\hat{\mathbf{T}}_c + \hat{\mathbf{T}}_a\right)p, \tag{1}$$

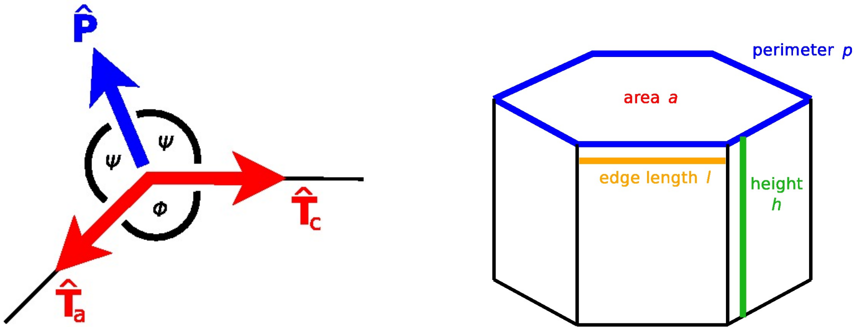

(a) (b)

Figure 3: *(a) Forces acting on vertices in the model. Tension forces act along cell edges in clockwise ($\hat{\boldsymbol{T}}_c$) and anti-clockwise ($\hat{\boldsymbol{T}}_a$) directions. Pressure forces bisect the angle ϕ between two edges ($\hat{\boldsymbol{P}}$). (b) Three-dimensional schematic of an epithelial cell, showing four key quantities used in the model; perimeter, p, area, a, height, h, and edge length, l.*

where C_L and C_P are constants related to the line tension and perimeter force, respectively, l_c and l_a are the lengths of the clockwise and anti-clockwise edges, and p is the total length of the cell perimeter. Large tension forces are created by large edge lengths and perimeters, and subsequently act to move neighbouring vertices closer together, reducing local edge lengths.

The pressure force is given by

$$\mathbf{P} = \left[C_A \frac{||a_t - a||^{n_1+1}}{(a_t - a)} + C_H H + C_D \frac{||\phi - \theta||^{n_2+1}}{(\phi - \theta)} \right] \hat{\mathbf{P}}, \qquad (2)$$

where C_A, C_H, and C_D are constants associated with the three components, a is the cell area, a_t is a 'target' area, H is the height-to-area ratio, θ is the average internal angle of the cell ($\theta = \pi(s-2)/s$ for an s-sided polygon), ϕ is the internal angle at the current vertex, and n_1 and n_2 are integer values, typically set to 2. The pressure force is thus dependent on cell area, height-to-area ratio, and local deformation. For further discussion of the meaning of each term, see Smith et al. (2011). The third term in (2) is important as it is a restorative force against cells becoming highly concave. During the migration

process, cells are likely to be under large stresses and strains. It is therefore possible that some cells, either those that are migrating or the surrounding cells, will become concave. *In vivo*, concave cells arise occasionally during migration when they are subjected to a lot of pressure, but they do not stay in this configuration for very long. A force is therefore included in the model to keep cells as near to a regular shape as possible.

The magnitude of the basic forces acting on each vertex can now be calculated. We need, however, to think further about the unit direction vectors $\hat{\mathbf{T}}$ and $\hat{\mathbf{P}}$ in equations (1) and (2), respectively, and how the force directions in Fig. 3(a) translate to the ellipsoid surface. The simplest approximation would be to use straight lines between vertices, however this would lead to forces acting directly through the interior of the ellipsoid, as in Fig. 4(a). A better approximation is for forces to act tangentially to the surface. To find the appropriate tangential direction for a given vertex, its neighbours are projected onto the tangential plane to the surface at the vertex, as in Fig. 4(b). Directions are then calculated on this plane, so that all forces act tangentially to the surface.

4 Equations of motion

To generate the equations of motion, we assume that inertial forces are negligible. This assumption is reasonable as viscous forces dominate in these types of systems (Odell et al., 1981). The equations of motion are given by

$$\mathbf{F}(\mathbf{x}, t) = \mu \frac{\mathrm{d}\mathbf{x}}{\mathrm{d}t}. \tag{3}$$

where $\mathbf{F}(\mathbf{x}, t)$ is a vector of the sum of the forces from Section 3 acting on the vertices, t is time, $\mathbf{x}(t)$ is the position vector of the vertices, and μ is the

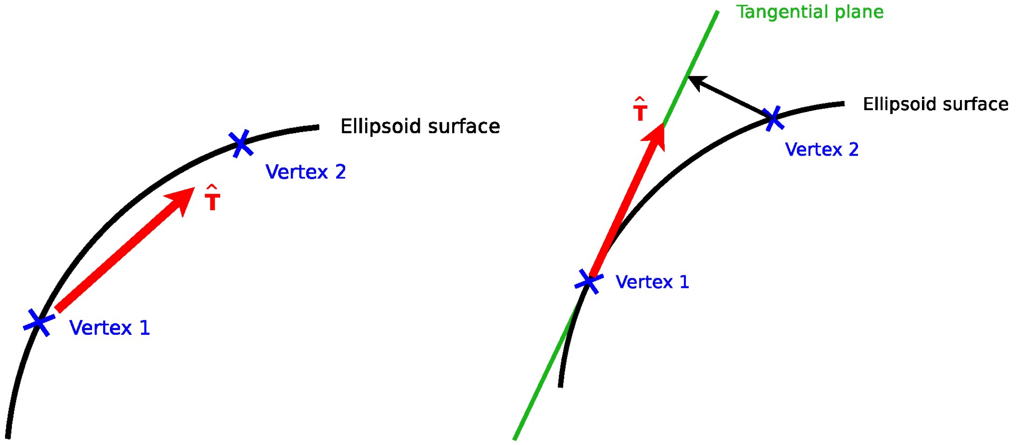

(a) (b)

Figure 4: *(a) Schematic of straight-line force direction from vertex 1 to vertex 2 on ellipsoid surface. (b) Equivalent tangential force direction from vertex 1 to vertex 2.*

viscous coefficient. The time derivative in (3) can be approximated by

$$\frac{\mathrm{d}\mathbf{x}}{\mathrm{d}t} = \frac{\mathbf{x}^{t+\Delta t} - \mathbf{x}^{t}}{\Delta t}, \tag{4}$$

where $\mathbf{x}$ is the vector of all vertex positions, and $\mathbf{x}^t$ represents the values of $\mathbf{x}$ at time t. This is the formal definition of a derivative in the limit $\Delta t \to 0$, and is a reasonable approximation for small values of Δt. We therefore obtain our discrete approximation to the equations of motion for every vertex,

$$\mathbf{x}^{t+\Delta t} = \mathbf{x}^{t} + \frac{\Delta t}{\mu}\mathbf{F}^{t}. \tag{5}$$

5 Constraining vertices to the ellipsoid surface

In equation (5) forces are applied freely in 3D, and vertices are therefore able to move away from the surface of the ellipsoid. We wish to constrain vertices

to the surface to maintain the ellipsoidal shape, so vertices must be mapped back at each iteration. For any point (x_1, y_1, z_1) in 3D space, the unit vector from the origin can be found via

$$(n_x, n_y, n_z) = \frac{(x_1, y_1, z_1)}{({x_1}^2 + {y_1}^2 + {z_1}^2)^{\frac{1}{2}}}. \tag{6}$$

The point at which this vector meets the surface of the ellipsoid must be found. In other words, the point

$$(\hat{x_1}, \hat{y_1}, \hat{z_1}) = (An_x, An_y, An_z), \tag{7}$$

is required, such that

$$\hat{x_1}^2 + \hat{y_1}^2 + \frac{\hat{z_1}^2}{c^2} = 1, \tag{8}$$

for some constant A. If this constant can be found, we will know the required point on the ellipsoid surface via equations (6) and (7). Combining (7) and (8) and rearranging for A, we obtain

$$A = ({n_x}^2 + {n_y}^2 + \frac{{n_z}^2}{c^2})^{-\frac{1}{2}}. \tag{9}$$

Fig. 5 demonstrates diagrammatically how a vertex is mapped to the ellipsoid surface. In reality it is ensured that vertices do not stray far from the surface, by keeping the value of Δt in (5) sufficiently small during simulations. This ensures that mapping to the ellipsoid has a minimal effect on the accuracy of the model.

6 Junctional rearrangements

As well as the force laws described above, elementary rearrangements between vertices are also included in the model. Junctional rearrangements occur *in vivo* between neighbouring vertices that are very close together. When

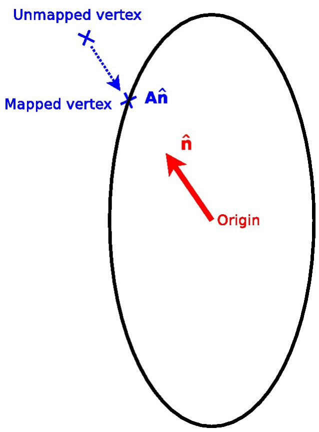

Figure 5: *Mapping a vertex to the ellipsoid surface. The vertex is mapped to the point on the ellipsoid surface which meets the straight line from the origin to the vertex.*

two vertices fall below a certain threshold distance, there is a probability of occurrence for each type of rearrangement. Junctional rearrangements are performed on the tangential plane to the surface at a given vertex (see Section 3), then projected back onto the surface of the ellipsoid.

6.1 T1 transitions

Fig. 6 explains a T1 transition diagrammatically. Vertices A and B are closer than the threshold distance, and rearrangement begins. Vertices C and D are created on a line that bisects the line AB perpendicularly. Cell 1 then reconnects to vertex C, cell 2 to vertex D, and cells 3 and 4 share both vertices C and D. In 3D, new vertices must be mapped back to the ellipsoid surface, as described in Section 5.

6.2 Vertex joining

As an alternative to performing a T1 swap, close vertices can simply be allowed to join up. The mid-point between the two vertices is used as the basis for a new vertex, and all previous connections are joined to it. This new

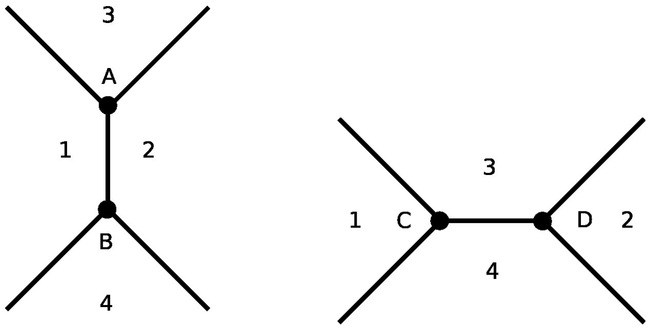

(a) (b)

Figure 6: *T1 swap for two close vertices. (a) Initially cells 1 and 2 share edge AB, which separates cells 3 and 4. (b) After the rearrangement cells 3 and 4 share edge CD, separating cells 1 and 2.*

vertex is then mapped to the ellipsoid surface. Vertex-joining permits the formation of rosettes, if a series of vertices fall below the threshold distance and join together. Fig. 7 shows a random initial 2D configuration, with close vertices then joined together. Several rosettes of five or more cells are clearly visible.

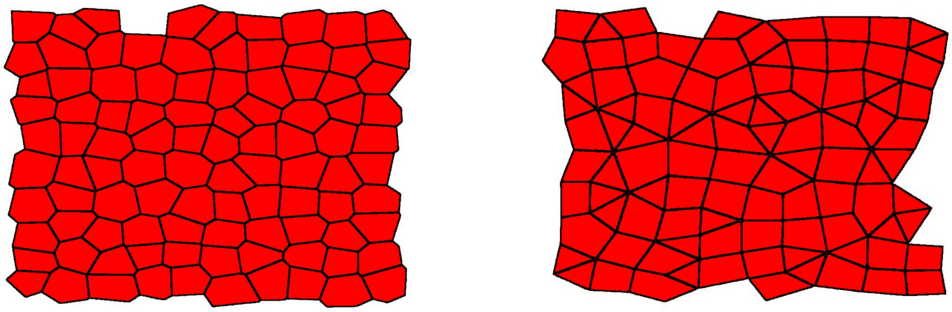

(a) (b)

Figure 7: *(a) Initial 2D cell configuration with (b) close vertices joined together.*

7 Cell growth and proliferation

Each cell i has an associated volume v_i, and is also given a growth speed g_i, drawn from a truncated normal distribution. Logistic growth is implemented according to

$$\frac{\mathrm{d}v_i}{\mathrm{d}t} = g_i v_i \left(1 - \frac{v_i}{V_T}\right),$$

where $v_i(t)$ is the volume of cell i and V_T is a target volume. Under this method, over time the average height of cells must increase, as this is the only way cells can grow without an increase in the surface area available to them. The height-to-area forces in (2) therefore also increase, leading to a higher outward pressure on each vertex. To compensate for this, the ellipsoid itself is allowed to grow over time, by increasing its radius linearly with time. Changing the radius means that (9), for mapping points back to the surface, must also be altered. When this is implemented, we observe that the mean height-to-area ratio of the cells is able to stay roughly constant, despite the overall growth in the ellipsoid. This is clearly dependent on the rates of growth of both the cells and the ellipsoid, but we are able to allow cells to grow during a simulation without significantly altering the pressure force.

Cell division in the model occurs at regular intervals, with the largest cell chosen to divide each time. As the numbers of cells at the start and end of migration are approximately known, the interval can be chosen accordingly. To implement cell division, initially two non-adjacent vertices of the cell are chosen at random. Two new vertices are then created on the the edges between each of the chosen vertices and their clockwise neighbours. These two new vertices are then joined to create a new edge dividing the mother cell into two daughter cells. Fig. 8 shows the implementation of mitosis.

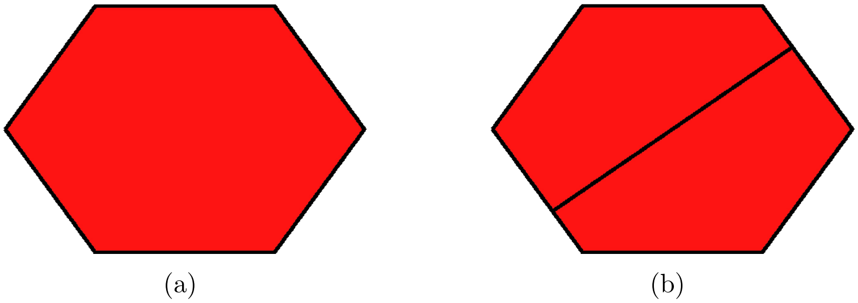

Figure 8: *Cell division in the vertex model. (a) Two non-adjacent vertices are chosen at random, and (b) two new vertices inserted on the edges between the chosen vertices and their clockwise neighbours.*

8 Simulating migration

It is now possible to simulate a growing embryo, with junctional rearrangements and cell proliferation. In order induce a few cells to migrate, and examine the effect this has on the system, the balance of forces acting on migrating cells must be altered. The migratory forces represent the dynamic internal changes that occur when a cell migrates. It has been observed that migrating cells often extend protrusions (Aman and Piotrowski, 2009) in the direction of migration. To simulate this a certain vertex is designated to be 'protruding', and the pressure force is increased at the protruding vertex. In the case of AVE migration it is known that cells migrate proximally, so we choose the proximal-most vertex of the migrating cell to be the protruding vertex.

9 Modelling the barrier

To incorporate the barrier between the epiblast and extra-embryonic endoderm into the model, vertices in the proximal half could simply be fixed, rendering them completely unable to move. However, this is somewhat unrealistic, as observations show that the proximal half is not completely static,

and junctional rearrangements are able to occur in this region. The proximal half appears to simply be less labile than the distal half, so we desire a way to represent this observation. It has also been observed that during the migration process actin expression is greater in the proximal half of the embryo, encompassing the complete surface of each cell. This may create a kind of tension force on the whole cell that makes it harder for its vertices to move. In the distal half of the embryo, meanwhile, actin is concentrated on cell edges. This tension force is already included as part of the basic model (Section 3). There is, however, a possible way to represent the actin shroud in the proximal half. In the equation of motion (3), there is a parameter μ that represents the local viscosity. This parameter can be chosen to be equal for all vertices, or can take a different value at each vertex. To simulate the expression of actin in the proximal half of the embryo, the viscous coefficient in that region is therefore increased.

References

T. Aegerter-Wilmsen, A. C. Smith, A. J. Christen, C. M. Aegerter, E. Hafen, and K. Basler. Exploring the effects of mechanical feedback on epithelial topology. *Development*, 137(3):499–506, 2010.

A. Aman and T. Piotrowski. Cell migration during morphogenesis. *Dev. Bio.*, 341:20–33, 2009.

R. Farhadifar, J.-C. Röper, B. Aigouy, S. Eaton, and F. Jülicher. The influence of cell mechanics, cell-cell interactions, and proliferation on epithelial packing. *Curr. Biol.*, 17(24):2095–2104, 2007.

K. P. Landsberg, R. Farhadifar, J. Ranft, D. Umetsu, T. J. Widmann, T. Bittig, A. Said, F. Jülicher, and C. Dahmann. Increased cell bond tension governs cell sorting at the Drosophila anteroposterior compartment boundary. *Curr. Biol.*, 19(22):1950–1955, 2009.

G. M. Odell, G. Oster, P. Alberch, and B. Burnside. The mechanical basis of morphogenesis. I. Epithelial folding and invagination. *Dev. Biol.*, 85(2): 446–462, 1981.

M. Rauzi, P. Verant, T. Lecuit, and P.-F Lenne. Nature and anisotropy of cortical forces orienting Drosophila tissue morphogenesis. *Nat. Cell. Biol.*, 10(12):1401–1410, 2008.

A. M. Smith, R. E. Baker, D. Kay, and P. K. Maini. Incorporating chemical signalling factors into cell-based models of growing epithelial tissues. *J. Math. Biol. (Accepted)*, 2011.

M. Weliky and G. Oster. The mechanical basis of cell rearrangement. I. Epithelial morphogenesis during Fundulus epiboly. *Development*, 109(2): 373–386, 1990.
